# Combining EEG-guided HD-tACS and Immersive Virtual Reality for Social Prediction Training in Congenital and Acquired Cerebellar Damage: A randomized sham-controlled trial protocol

**DOI:** 10.1371/journal.pone.0353955

**Published:** 2026-08-04

**Authors:** Andrea Ciricugno, Renato Borgatti, Zaira Cattaneo, Francesco Di Russo, Marco Iosa, Viola Oldrati, Romina Romaniello, Claudia Salera, Libera Siciliano, Gaetano Tieri, Cosimo Urgesi, Maria Leggio

**Affiliations:** 1 IRCCS Mondino Foundation, Pavia, Italy; 2 Department of Brain and Behavioral Sciences, University of Pavia, Pavia, Italy; 3 Department of Human and Social Sciences, University of Bergamo, Bergamo, Italy; 4 LIFE - Institute for research and treatment - Santa Lucia IRCCS, Rome, Italy; 5 Department of Movement Human and Health Sciences, University of Rome “Foro Italico”, Rome, Italy; 6 Department of Psychology, Sapienza University of Rome, Rome, Italy; 7 Scientific Institute, IRCCS E. Medea, Bosisio Parini (LC), Italy; 8 Department of Human Sciences, Link Campus University, Rome, Italy; 9 Department of Law and Digital Society, Unitelma Sapienza University, Rome, Italy; 10 Department of Human and Social Sciences, Universitas Mercatorum, Rome, Italy; PLOS: Public Library of Science, UNITED KINGDOM OF GREAT BRITAIN AND NORTHERN IRELAND

## Abstract

The cerebellum is increasingly recognized for its crucial role in high-level cognitive processes, particularly in the domain of social cognition and action prediction. Disruptions in cerebellar circuits can lead to significant impairments in anticipating others’ intentions and behaviors, affecting daily social interactions. This randomized, double-blind, sham-controlled trial protocol investigates the combined effects of personalized cerebellar High-Definition transcranial Alternating Current Stimulation (HD-tACS) and Immersive Virtual Reality (IVR) training on social prediction abilities. The study involves two clinical populations: adolescents and young adults with congenital cerebellar malformations (CM) and adults with acquired neurodegenerative cerebellar atrophy (CA). Participants undergo eight daily sessions of IVR training designed to enhance internal models of social events through interactive scenarios. Simultaneously, they receive either active or sham HD-tACS delivered at their Individual Gamma Frequency (IGF), determined via baseline EEG. The primary outcomes include behavioral responses to context-based social prediction tasks (action intentions, emotions, and personality traits). Secondary outcomes encompass electrophysiological measures, neuropsychological functioning, and adaptive behavior. By integrating neuromodulation with embodied virtual experiences, the project aims to facilitate cerebello-cerebral plasticity and provide a novel transdiagnostic rehabilitative approach for socio-cognitive impairments. The present protocol has been registered on ClinicalTrials.gov (NCT07500103).

## Introduction

Cerebellar alterations have been linked not only to sensorimotor deficits but also to a constellation of cognitive, social, and affective dysfunctions known as the Cerebellar Cognitive Affective Syndrome (CCAS) [1–4]. This syndrome affects both children and adults with congenital or acquired etiologies [5–7]. Indeed, the cerebellum, particularly its posterior sectors, is increasingly recognized as a critical hub within distributed neural circuits underlying cognitive and social functions, through bidirectional connections with associative and paralimbic cerebral areas [8–13]. A leading hypothesis posits that the cerebellum supports social cognition by generating internal sequencing predictions based on input from cerebral mentalizing regions, while signaling errors when mismatches occur between anticipated social events and ongoing behaviors [14,15]. Analogous to its role in the sensorimotor domain [16,17], the cerebellum modulates higher-order cortical activity in the social domain by maintaining internal models of social action. This mechanism enables optimized feedforward control, which is essential for the fluid and automated execution of social interactions [18,19]. Consequently, cerebellar dysfunction disrupts these internal models, impairing prediction and resulting in altered social behavior [7,20].

Despite these neuroscientific advances, rehabilitation strategies for CCAS remain significantly under-researched [7,21,22]. However, preliminary evidence supports the efficacy of Virtual Reality (VR) training for enhancing social cognition in cerebellar patients [7,21]. Specifically, children and young adults with congenital cerebellar malformations demonstrated greater benefits from a VR intervention tailored to social prediction abilities compared to a control non-social VR motor training. This advantage was reflected not only in improved social prediction performance but also in the generalization of training effects to secondary neuropsychological outcomes, such as theory of mind and inhibitory control [21]. By providing embodied sensorimotor experiences within complex, interactive social scenarios, VR represents a highly promising tool for rehabilitation [23,24].

Growing evidence suggests that Non-invasive Brain Stimulation (NIBS), either alone or combined with behavioral interventions, holds promise for mitigating social and cognitive impairments in clinical populations. Techniques such as Transcranial Magnetic Stimulation (TMS) and Transcranial Electrical Stimulation (tES) have been successfully applied to the cerebellum in healthy individuals, advancing our understanding of its role in social cognition (for reviews see [25–27]). Building on this foundation, several studies have employed repeated sessions of cerebellar neuromodulation to enhance treatment effects and alleviate socio-affective symptoms, reporting positive outcomes across various neurological, neurodevelopmental, and psychiatric conditions [26,28,29]. While therapeutic applications in cerebellar pathology have traditionally targeted motor recovery (e.g., [30,31]), recent research highlights a novel potential for social cognition. Notably, Oldrati et al. [32] demonstrated that a single session of cerebellar anodal transcranial direct current stimulation (tDCS) improved the use of contextual information to predict others’ mental states in adolescents and young adults with cerebellar malformations. This finding underscores the feasibility and therapeutic potential of such approaches in this specific population.

Among tES techniques, transcranial Alternating Current Stimulation (tACS) has recently emerged as a powerful tool for modulating cerebellar functions (see [33]). tACS delivers weak alternating electrical currents at specific frequencies to the scalp, modulating neuronal activity and behavior [34]. While initial hypotheses focused on neural entrainment—aligning endogenous brain rhythms with the external current [35]—recent evidence emphasizes complex interactions between the applied sinusoidal current and ongoing neural activity [36]. In particular, these tACS effects appear to be highly state-dependent, meaning that the stimulation’s impact is critically modulated by the endogenous power and phase of the target oscillation at the moment the current is applied [37]. Given that disrupted oscillatory patterns characterize several neurological disorders, tACS offers a mechanism to restore physiological rhythms. When applied to the cerebellum, tACS has proven effective in promoting plasticity and improving motor performance in both healthy individuals [38–40] and patients with cerebellar disorders [41]. Crucially, stimulation in the gamma range (30–80 Hz)—corresponding to the basal firing rate of Purkinje cells, the main cerebellar output neurons—has consistently shown efficacy in modulating cerebellar motor functions [33]. These findings strongly encourage the exploration of cerebellar gamma tACS as a low-cost clinical tool for treating non-motor impairments.

Building on this evidence, the present protocol aims to investigate the combined effects of a neuromodulation protocol—designed to facilitate cerebello-cerebral plasticity—and Immersive Virtual Reality (IVR) training targeting internal models of social events. The study involves early adolescents and adults with congenital non-progressive cerebellar malformations, as well as adults with neurodegenerative cerebellar disorders. To maximize efficacy, the intervention leverages previous findings suggesting that weak exogenous electrical stimulation effectively modulates neural oscillations only when matching endogenous rhythms [36]. Consequently, rather than overriding intrinsic dynamics, we aim to enhance them using a personalized High-Definition (HD)-tACS protocol. Specifically, stimulation will be delivered at each participant’s Individual Gamma Frequency (IGF). We hypothesize that coupling this personalized stimulation with IVR-assisted social prediction training will potentiate treatment outcomes by promoting brain plasticity and boosting cognitive learning. Furthermore, including both congenital and progressive conditions allows for the evaluation of distinct pathological models. While congenital malformations impact neural development and the functional organization of circuits during highly plastic early stages, neurodegenerative disorders affect fully established networks. Comparing these groups offers a unique opportunity to investigate the plasticity of cerebello-cerebral social circuits, revealing specific anatomo-functional associations and distinct recovery potentials. Ultimately, this transdiagnostic approach may identify critical therapeutic targets for improving socio-cognitive abilities in other conditions linked to cerebellar dysfunction, such as Autism Spectrum Disorder, dementia, and mood disorders [42–44].

## Method

### Study design

**Fig 1:**
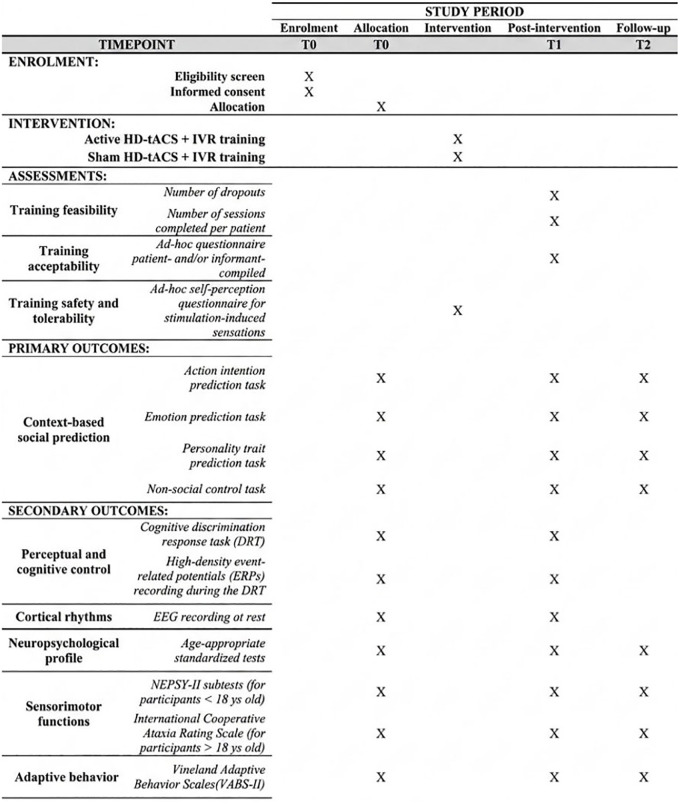
SPIRIT Schedule of enrolment, interventions and assessments.

We present the study protocol of the project “SINCRO: Spatiotemporal entrainment as Innovative Neuromodulation targeting Cerebello-cerebral circuits for enhancing Rehabilitation Outcomes of cognitive and social skills in progressive and acquired cerebellar diseases” (Italian Ministry of Health, RF-2021–12374279). The study employs two arms of a randomized, double-blind, sham-controlled, pre-post-test trial (RCT) with 30 adolescents and young adults with congenital cerebellar malformations (CM) and 30 adults with degenerative cerebellar atrophy (CA), respectively (see below for sample size calculation). Eligible patients are contacted by medical doctors and those willing to participate are enrolled. Participants undergo eight daily (1 hour) sessions of an IVR training of social prediction abilities, receiving either active or sham cerebellar IGF HD-tACS. Each participant is assigned randomly to either the active or sham group in a 1:1 allocation ratio, using a computer-generated blocked randomization process carried out by independent personnel not directly engaged in the study. See Fig 1 for the SPIRIT Schedule of enrollment, interventions, and assessments.

Randomization is stratified according to age and IQ stratification factors. In particular, for patients with cerebellar malformations, we consider 2 levels for age, corresponding to 12–17 and>=18 years old, and 3 stages for cognitive level consistent with, respectively, absence of intellectual disability (IQ > 80), from borderline intellectual functioning to mild intellectual disability (80 >=IQ >=61) and from mild to moderate intellectual disability (60 >=IQ >=50). Doing so, six blocks are generated and within each block 5 patients should be enrolled to achieve and overcome the established sample size. A similar approach is used for patients with degenerative cerebellar atrophy, but only age is stratified with 2 blocks corresponding to 18–35 and 36–68 years old, with 15 patients per block. First, participants are allocated to a block depending on the stratification variables. Second, patients are assigned to one of the two interventions according to a specific permuted sequence.

**Fig 2:**
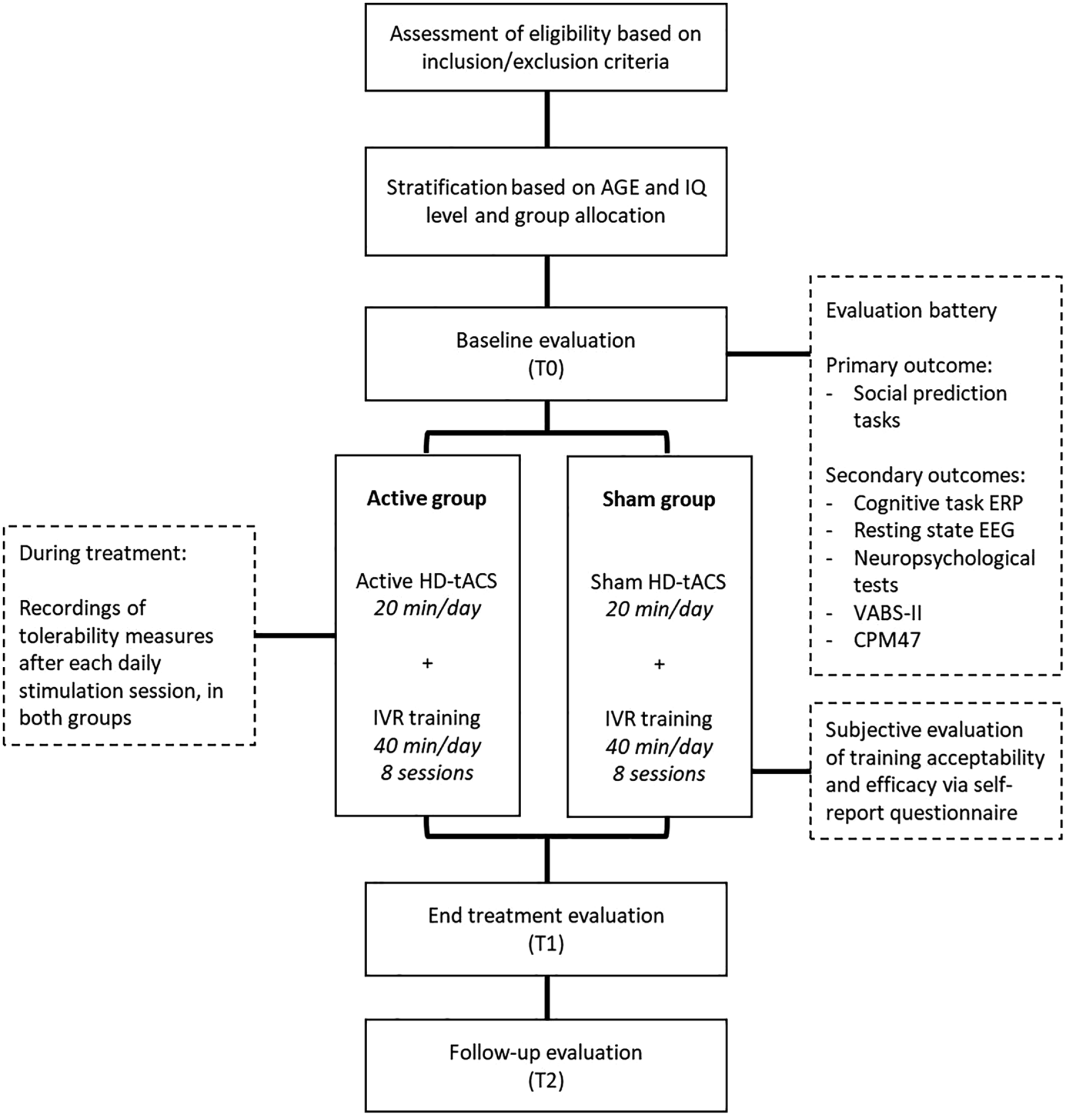
Flow chart of patient inclusion.

Staff conducting pre/post assessments, personnel leading the IVR training and researchers analyzing data are blinded, whereas the personnel administering the stimulation and the project administrators are not. The personnel administering the stimulation is the only person in the room aware of the correspondence between a specific code and stimulation protocol and leaves when the stimulation (either active or sham) is over, thus preventing any influence on training administration. Of note, the electrode placement procedure (see EEG-guided HD-tACS section) is identical for both sham and active stimulation conditions, which helps maintain blinding of both participants and study personnel to treatment allocation, ensuring that the personnel conducting the training remain unaware of the treatment allocation. Unblinding should only occur in rare cases where knowledge of the treatment is crucial for managing the patient, such as in the case of adverse events. Assessments occur at baseline (T0; from one to two days before the beginning of the training), post-treatment (T1; from one to two days after the end of the training), and two months follow-up (T2). All primary and secondary outcomes are assessed at each time point, except for questionnaires on stimulation-induced sensations, which are evaluated after each daily stimulation session, and behavioral, electroencephalographic (EEG) event-related potential (ERP) measures during a cognitive task, which are administered solely at T0 and T1 (see Fig 2).

Both RCT arms are structured as follows. Prior to the beginning of the training (T0), the procedure requires the administration of all primary and secondary outcome measures. Additionally, resting-state EEG is recorded and analyzed to calculate the IGF, which is used as each patient’s specific stimulation frequency delivered through HD-tACS during the IVR training. The treatment is conducted at IRCCS Mondino Foundation for patients with cerebellar malformations and at Ataxia Laboratory at IRCCS Santa Lucia for patients with degenerative cerebellar atrophy. Recruitment began on January 15, 2025 – following Ethics Committee approval but while the formal trial registration was pending due to administrative personnel transitions at the IRCCS Santa Lucia. This decision was taken to meet the stringent project reporting deadlines, which carried non-compliance penalties. Recruitment is expected to conclude on December 14, 2026. Data collection will continue through the follow-up period, ending two months after the last participants are enrolled, in February 2027. Results are expected to be available by April 2027, a few months after data collection is completed. The authors confirm that all ongoing and related trials for this intervention are registered.

### Patients selection

We recruit patients showing cerebellar malformations and variable phenotypes and patients affected by degenerative cerebellar atrophy. Inclusion criteria for the CM patients include: documented malformations confined to cerebellum confirmed by a 3T brain MRI scan; age ranging from 12 to 32 years old; IQ >= 50; absence of extra-cerebellar malformations on conventional brain MRI scan. Inclusion criteria for CA patients include: evidence of diffuse cerebellar atrophy; more than 6 months of illness; IQ >=75; absence of any cortical lesion on conventional brain MRI scans. The exclusion criteria for both groups include: presence of severe motor and visual impairments, as well as neurodevelopmental (i.e., autism), neurological or psychiatric disorders that could interfere with task execution and protocol compliance; presence of any contraindication for tACS. Age-appropriate Wechsler scales and Coloured Progressive Matrices (CPM47) will be administered to assess intellectual level.

### Outcomes

#### Primary outcome.

The primary outcomes comprise the behavioural responses to three computer-assisted, context-based social prediction tasks and a non-social control task. These tasks are designed to assess the ability to use contextual information to infer and predict others’ mental states or traits, including action intentions, emotional states, and personality traits. All tasks share the same procedure: each trial presents a priming stimulus (a situational context) followed by an ambiguous target stimulus. In half of the trials, context and target are congruent, confirming the prediction; in the other half they are incongruent, contradicting it. Participants then select between two possible interpretations of the mental state or trait conveyed by the target. In previous studies using similar tasks [45], we found that the preceding situational context influenced participants’ ability to interpret the target: accuracy was higher and reaction times were shorter in congruent than in incongruent trials, indicating that participants used contextual information to infer mental states.

- Action intention prediction task: Contexts are photographs of dining tables (e.g., with empty or filled glasses), followed by an image of a hand reaching toward an object (e.g., a bottle). Pictures are taken before hand–object contact, capturing the pre-shaping of the hand during the reach-to-grasp phase. Participants infer whether the action intends to use the object (e.g., pouring) or to move it (e.g., putting it away).
- Emotion prediction task: Contexts are positive or negative social scenarios (e.g., a party, a car accident), followed by morphed facial expressions representing happiness or fear. Participants indicate which emotion is expressed.
- Personality trait prediction task: Contexts depict morally acceptable (e.g., helping an elderly person) or unacceptable behaviors (e.g., stealing, polluting), followed by a target face from a validated database [46] normatively rated as trustworthy or untrustworthy. Participants judge whether the face represents a trustworthy person.
- Non-social control task: Contexts are indoor or outdoor environments, followed by blurred images of natural (e.g., a tree) or artificial objects (e.g., a ball) from an existing database [47]. Participants classify the target as natural or man-made.

### Secondary outcomes

Secondary outcomes include electrophysiological measures of anticipatory control, perception, and attention, neuropsychological functioning, adaptive behavior, and fluid intelligence.

- A cognitive discrimination response task (DRT): Used as an index of perceptual and cognitive control, such as inhibition, a core executive function supporting social cognition. Participants respond quickly to target stimuli and withhold responses to non-target stimuli.
- High-density event-related potentials (ERPs) recording: ERPs are measured during the DRT, to examine pre- and post-stimulus activity, capturing reactive sensory-motor processes, proactive motor preparation, and cognitive anticipation.
- Cortical rhythms: EEG recordings are collected at rest (5 minutes) and during the DRT (15 minutes) to assess changes in delta, alpha, beta, and gamma power across time points, focusing on regions linked to socio-cognitive functions.
- Neuropsychological profile: Age-appropriate standardized tests are administered. For participants ≤16 years, the NEPSY-II battery is used, covering six domains (Attention/Executive Function, Language, Memory/Learning, Social Perception, Visuospatial Processing, Sensorimotor Skills). For participants >16 years, equivalent measures are administered, including the Albert Test [48], Stroop Test [49], Token Test [50,51], Rey–Osterrieth Complex Figure (Copy and Recall) [49], Reading the Mind in the Eyes Test [52], Faux Pas Test [53], and WAIS-IV Block Design [54]. Furthermore, for patients ≥ 18 years, the Cerebellar Cognitive Affective/Schmahmann scale (CCASS), recently validated for the Italian adult population [55] is administered.
- Sensorimotor functions: For participants < 18 years, subtests of the Italian version of the NEPSY-II (i.e., Finger Tapping, Manual Motor Sequences, Imitating Hands Position) are administered. For participants ≥ 18 years, the International Cooperative Ataxia Rating Scale (ICARS [56]) is used.
- Vineland Adaptive Behavior Scales, Second Edition (VABS-II [57]): a tool designed to assess adaptive behavior in individuals from birth to age 90 years, including open-ended questions to gather in-depth information. This scale is used to assess adaptive skills across 4 domains: communication, daily living skills, socialization and motor skills. It provides 5 scores for each domain and a total score on a 100 ± 15 distribution.
- Coloured Progressive Matrices (CPM47 [58]): A non-verbal measure of reasoning and fluid intelligence, consisting of 47 items requiring identification of the missing element in visual patterns.

Primary outcomes are assessed at all time points (T0, T1, T2). Among secondary outcomes, the DRT and EEG recordings are conducted at baseline (T0) and immediately after training (T1). Neuropsychological evaluation, VABS-II, and CPM47 are administered at each time point.

### Safety and tolerability measures

To assess safety and tolerability to the HD-tACS protocol, patients are asked to complete, at the end of each stimulation session, a customized self-perception questionnaire to examine the stimulation-induced sensations (including: tingling, burning, pain, mouth taste, visual sensations like flashes, eyelid movements, others) through a series of Likert scales.

### Feasibility and acceptability of the training

The feasibility of the training is assessed by considering i) the number of dropouts and ii) the number of sessions completed per patient. These values, expressed as percentage and mean percentages, are extrapolated at T1. Furthermore, acceptability is assessed by asking patients’ and their family’s subjective evaluations of training accessibility and efficacy. The questionnaires are adapted from the study of Butti and colleagues [21]. Questions like “I find it difficult to motivate my child for doing the training”/ “I find it difficult to start the training”; or “I would suggest this training to other people I know”/ “I think other people I know would enjoy doing this training” are rated from 1 (completely disagree) to 5 (completely agree). Responses are reversed in the negative items, so that higher scores suggest more positive evaluation/higher acceptability. Lastly, three questionnaires are administered to validate the sense of embodiment and presence in the virtual scenarios (embodiment questionnaire [59]), as well as their usability (USEQ22, Gil- Gomez et al., 2017 [60]; NASA-TLX, [61]).

### EEG-guided HD-tACS

**Fig 3:**
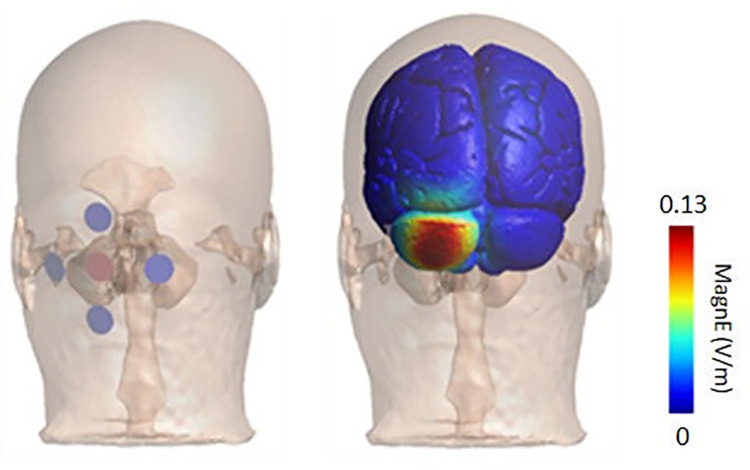
HD-tACS electrodes montage and E-field Simulation. Representation of the estimated electric field induced by cerebellar IGF HD-tACS using five 3,14 cm^2^ round electrodes, the active electrode placed over the left posterior cerebellum (centered 2 cm below and 3 cm to the left of the inion) while the four reference electrodes will be positioned in a circle and equidistant from the center (4 cm). The head model was created using fine element modeling on T1- and T2-wighted MRI images of an exemplary subject resulting in a high-resolution tetrahedral head mesh model containing 8 tissue types. Electrodes of 1 mm thickness were simulated with 2 mm thick of conductive gel. All tissues were treated as isotropic. The electrical field E was determined by taking the numerical gradient of the electric potential.

The tACS is performed by using a CE-marked DC stimulator device (Starstim ®, Neuroelectrics, Barcelona, Spain). It consists of a multi-focal tES-EEG device with 8 channels. The current is delivered through five 3.14 cm^2^ ring electrodes. The active electrode is placed over the left (i.e., centered 2 cm below and 3 cm to the left of the inion, Iz) cerebellar hemisphere, as advised in a computational electric field modeling study [62], while the four reference electrodes are positioned in a circle and equidistant from the center (4 cm). This montage was chosen based on previous studies and a simulation model of the electric field distribution and magnitude generated by the applied montage performed with SimNIBS 4.1 [63,64] with standard conductivity values (see Fig 3). tACS is delivered over the left cerebellar hemisphere, based on a previous large-scale neuroimaging study demonstrating stronger and more effective connectivity between left cerebellar regions sensitive to mentalizing and the right cerebral mentalizing areas, compared to the opposite (right cerebellar–left cerebral) configuration [65]. The stimulation intensity is set to 1 mA peak-to-peak, and this value is gradually reached with a ramping-up phase of 30 secs. At the end of the stimulation time, current gradually fades out with a similar 30-sec ramp-down phase. This intensity was specifically selected to account for the thinner scalp and lower cranial resistance of younger participants [66], supported by previous evidence demonstrating that adolescents and adults exhibit comparable responses to 1 mA stimulation [67]. EEG data are recorded at 500 Hz sampling rate, from 19 Ag/AgCl electrodes (C3, C4, Cz, F3, F4, F7, F8, Fp1, Fp2, Fz, O1, O2, P3, P4, Pz, T3, T4, T5, T6) in a 10/10 system, using the right earlobe for both ground and reference. After recording, EEG data are exported to a format compatible with Matlab® (The Mathworks Inc., 2023) and processed within the EEGLAB signal processing toolbox and custom Matlab® scripts. Continuous EEG data are filtered using a 0.5-Hz high pass and then a 100-Hz low pass FIR filter. For each electrode, the power spectral density (PSD) is estimated using Welch’s method. The specific frequency within the gamma band (ranging from 30 to 80 Hz), observed during rest and exhibiting the greatest power, is labeled as the IGF. This frequency then serves as the stimulation frequency tailored to each individual patient. The HD-tACS is applied for 20 minutes before the beginning of the training, every day of the treatment. In the sham condition, after the initial ramping-up, the stimulation is switched off. This procedure allows participants to feel the characteristic tingling sensations in the vicinity of the electrodes for a brief period of time, which enhances the plausibility of the sham condition.

### Immersive Virtual Reality training

#### Virtual scenarios.

The virtual scenarios and objects were developed in Blender 3D (https://www.blender.org/) and implemented in Unity Game Engine (https:/www.unity.com/). The scenarios were designed to represent realistic settings and situations appropriate for the age groups of participants (adolescents and adults). In detail, the scenarios for adolescents presented a school library and an outdoor playground, while the scenarios for the adults included a supermarket and a restaurant.

**Fig 4:**
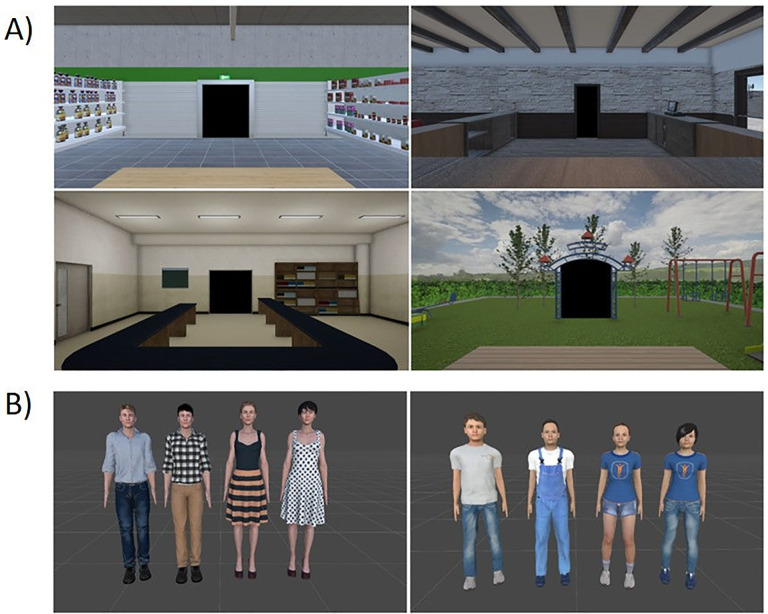
Immersive Virtual Reality (IVR). A) Virtual scenarios for adult patients (top left panel: supermarket scenario, top right panel: restaurant scenario) and adolescent patients (bottom left panel: library scenario, bottom right panel: playground scenario); B) Virtual avatars for the adults’ (left panel) and adolescents’ (right panel) scenarios. This figure was created by the authors and also featured in Siciliano et al., [68].

In addition, a practice scenario was created for each age group, to be presented at the beginning of the first training session only. The layout, objects and trial structure of this scenario are identical to those in the experimental scenarios, but the scene does not contain any details to identify the virtual space; the virtual objects are simple 3D geometric figures, and the avatars are represented as grey shapes without distinct features. All scenarios have a similar layout that included a surface (a counter) where the virtual objects were placed, which is located in front of the participant, and a door at the far frontal end of the room/space (Fig 4A).

Four virtual avatars were created for each set of scenarios (adolescent and adult versions) with MakeHuman (http://www.makehumancommunity.org/) and iClone 3D (www.reallusion.com) software. The avatars’ body size and features were age-matched with age of the two target groups (i.e., adolescents and adults). In both sets, two avatars were male and two were female, and all avatars differed in certain characteristics such as hair color, hairstyle, and clothing (Fig 4B). All avatars were animated in Unity Game Engine with the animation of naturalistic walking. The scenarios will be presented to the participants via a Meta Quest 2 head-mounted display (HMD) with RGB LCDs with 1832x1920 pixels resolution, refresh rate at 120 Hz, the field of view of 110° (diagonal FOV) and 6 degrees of freedom. Participants will interact with the content of the scenarios via Meta Quest 2 hand-held game controllers for both hands.

### Procedure

In all the scenarios, participants observe the virtual scene from the first-person perspective. The only visible part of their virtual body is the hands, which move synchronously with participants’ hand movements performed with the game controllers. In the scene, participants are located above a chair in front of the counter that is reachable with a forward hand movement. At the onset of each trial (activated by the participants via pressing the A key on the right controller), three different objects appear on the counter in front of the participant. Together with the appearance of the objects, one of four avatars enters the scene through the door located in the far front end of the scene. The avatar approaches the counter by walking at a constant speed and grasps one of the objects. In the first four trials, participants are instructed to observe the avatars and the objects without performing any movements. After that, participants are asked to anticipate which object the avatar would grasp and to grasp that object themselves before the avatar does. Once either the avatar or the participant grasps one of the objects, the avatar and the objects disappear and the trial is concluded.

The virtual objects in each scenario belongs to one of three categories: in the supermarket, the categories include packaged foods, beverages and household products; in the restaurant, the objects belong to the categories of foods, beverages and sauces; in the library, the objects are books, pencil cases and stationery objects; in the playground, the objects are stuffed toys, books and flowers; in the practice scenario, the objects are cubes, cylinders and spheres with sizes matching those of the objects used in the other scenarios. In each scenario, three avatars show a preferred category of objects that they grasp in all trials, while the fourth avatar picks the objects randomly from any category. The avatar-object category mapping are pseudorandomized and differ across the eight training sessions; moreover, in addition to the object preferences, avatars’ clothes change between the scenarios, in order to prompt the participant to focus on the participant’s identity, rather than simply associating each object with cloth colour. Each of the experimental scenarios include 44 trials that last approximately 7 minutes in total. Within each training session, the two scenarios developed for the age-group are presented in two different blocks, the order of which is counterbalanced across participants. Thus, each training session lasts for approximately 30 minutes.

Before starting the experimental scenarios, participants sit on a chair in a lab with enough surrounding area to safely perform the reaching movements. They are instructed to sit with their back against the back of the chair and their arms placed on the armrests, after which they put on the HMD and take the controllers in each hand. Then, only at the beginning of the first training session, the practice scenario is launched, and the participants could first take a few moments to gain confidence with the virtual environment, performing hand movements, looking around, and describing what they see. These actions are also performed in the beginning of each training session and are necessary to create a sense of embodiment and presence in the virtual environment [69]. In the practice scenario, the structure and the aim of the task are explained, that is participants are instructed how to grasp the virtual objects, how to start the trials, and what the main goal of the task is (predicting the avatars’ preferences in picking the objects and grasping that object before the avatar). Crucially, participants are informed that all avatars may show a preference for one category of objects. If the participants grasps the correct object (i.e., the target object of the avatar), a high-pitched sound is delivered via the HMD speakers; otherwise, a low-pitched sound marks erroneous responses. Participants then perform several trials of the practice scenario until they are comfortable with the grasping movement and the procedure. After that, they perform the two experimental scenarios that will be presented in a counterbalanced order.

### Sample size calculation

Using the GPower 3 software [70] with the “as in SPSS option”, we estimated that, with a mixed design between (group factor: active group vs. sham group) – within (Time points: T0, T1, T2, numerator DF = 2), a sample of 15 subjects per group allows detecting moderate to large effects (f (U)=0.428), based on previous study of the effects of cerebellar electrical stimulation on action prediction tasks in patients with cerebellar malformation [32] with a power of 0.80, setting a significance threshold of 0.05. Hence, 30 CM patients and 30 CA patients will be recruited.

### Statistical analyses

Data analysis will be conducted using Jamovi 2.6. Demographic and clinical variables of the two groups of patients will be inspected through descriptive statistics. Independent sample t test (two-tailed) and χ2 will be used to assess the differences between the experimental and control training groups at baseline for continuous and categorical variables, respectively, thus allowing us to verify successful randomization.

Normality of data distribution will be assessed using the Shapiro-Wilk test, whereas the Levene’s test will be used to assess the homogeneity of the variances. Parametric statistical tests will be used if data will result normally distributed, with proper corrections if the assumption of variance homogeneity was violated.

To evaluate intervention efficacy, an intention-to-treat analysis approach will be adopted, including in the analyses all the participants who are allocated into the two study groups and complete at least one evaluation, irrespective of adherence to treatment (i.e., if they do not complete all the training sessions) and evaluations (i.e., if they do not complete evaluations at all time points). No imputation of missing data will be used considering the limited sample size and observation points.

Linear mixed models (LMMs) will be used to treat data considering each primary outcome measure as a dependent variable in separate models. Treatment (active vs. sham HD-tACS as between-subjects variable), time (T0, T1, and T2) and their interactions will be entered as fixed factors, while patients included will be entered as random factors. To take into account a-priori sample differences (even if not statistically significant), we will also consider the age and IQ of participants as possible covariates in follow-up analyses. If one of these covariates will result statistically significant, data will be stratified according to homogeneous values of the significant variable and a secondary analysis will be conducted on homogeneous subgroups for clarifying the effect of the covariates, correcting the alpha level to avoid the possible inflation of first type error. A significance threshold of *p* = 0.05 will be set for all analyses. Post-hoc comparisons will be performed only on statistically significant factors to identify the significant difference between the levels of the independent variables, applying multiple correction methods for type I error. In case of not normally distributed data, the first approach will be the adoption of techniques to normalize data such as the use of z-scores or percentage points; if these kinds of normalization fail, data will be summarized and analyzed by using non-parametric statistics. Data will be reported in terms of median values and quartiles. Instead of LMMs, Kruskal-Wallis analysis for between-subject comparisons will be adopted, together with Friedman’s analysis to compare within-subject parameters. All the analyses will be performed at the end of all T1 evaluations, and no interim analyses will be performed.

### Data safety and management

The project entails the processing and storage of pseudonymized data, which are securely archived in a centralized repository situated on the servers of the study sponsors. Questionnaire scores and neuropsychological test results do not include any personal information regarding the participants, who are distinguished solely by an alphanumeric code. Any records containing names or other personal identifiers, including informed consent forms, are stored separately from the study records identified by code number. Access to the archives is strictly controlled and granted solely through a rigorous authentication procedure, ensuring appropriate usage rights are maintained.

### Ethics and dissemination

Approval for this study was granted by the local ethics committees (Comitato Etico Pavia, Prot. N. 0025455/23, approved on May 15, 2023; Comitato Etico Fondazione Santa Lucia Roma, Prot. CE/2023_001, approved on January 31, 2023). Written informed consent is obtained from the parents or legal guardians of minors, and directly from adult participants. Minors as well are asked to give their assent for participation. Subjects are discontinued in the case of serious adverse events. Important protocol modifications will be reported to the Italian Ministry of Health, and approved by the Ethics Committees.

Patients and the public were not involved in the design of the study nor are involved in its conduct, while patients’ associations will be involved in the dissemination plans of the results. The outcomes of this study will be shared with the involved physicians, referring practitioners, patients, and the broader medical community. Presentation of results is planned at academic and/or clinical conferences. Furthermore, the study aims to produce at least one peer-reviewed publication of quantitative findings in a reputable national or international medical journal. Reporting of this RCT will adhere to CONSORT guidelines [71] to ensure transparency and completeness in the presentation of results. The authors will adhere to principles of transparency and reproducibility in research by following the procedures presented in this study protocol and by sharing research material and anonymized dataset of this study in public repositories (https://osf.io/).

## Discussion

The role of the cerebellum has been radically redefined in recent decades, extending its functional domain well beyond motor control. It is now widely acknowledged as a critical node within the distributed neural networks subserving cognitive and social processing [72,73]. However, despite this increasing neuroscientific knowledge, only a limited number of studies have addressed the rehabilitation of CCAS so far [7,21,22]. One of the first and few attempts in this direction utilized VR; specifically, a recent study on patients with cerebellar malformations showed that a VR intervention targeting social prediction was effective not only in enhancing these functions but also in producing effects generalizable to standard measures of theory of mind and inhibitory control [21]. A promising and complementary avenue for the further potentiation of these skills is represented by cerebellar neuromodulation. While neuromodulation has already demonstrated feasibility and safety across various neurological and psychiatric populations, even reducing social-affective symptoms in some instances [26,28,29], its application in patients with primary cerebellar damage has remained focused almost exclusively on motor recovery [26]. Despite this traditional focus, preliminary data suggest that cerebellar neuromodulation could be a key modality for boosting social prediction abilities [32], highlighting the therapeutic potential of integrating such innovative approaches to rehabilitate social skills in this population.

Based on this theoretical framework and the encouraging preliminary findings, the aim of the present protocol is to present a novel, multimodal therapeutic approach specifically designed to address the cognitive and social deficits characterizing patients with cerebellar alterations. Crucially, this protocol advances previous methodological approaches through specific innovations regarding both the behavioral and neuromodulatory components. Unlike previous protocols (e.g., [21]), this protocol employs an IVR system to maximize patient engagement and the sense of presence, promoting a higher degree of embodiment. By simulating realistic social scenarios, IVR enhances the ecological validity of the training, thereby facilitating the transfer of acquired social prediction skills to real-life interactions. Concurrently, regarding the neuromodulation component, this protocol moves beyond traditional approaches (e.g., TMS or tDCS) by utilizing tACS. While cerebellar tACS has been applied almost exclusively to investigate and enhance motor functions so far (see [33]), it offers a unique advantage. Instead of merely exciting or inhibiting neuronal excitability (as for instance tDCS), tACS allows for the modulation of the intrinsic oscillatory behavior of the target area. This mechanism is critical for promoting neuroplasticity within the cerebro-cerebellar circuits, as it can exert effects on distal cortical regions functionally connected to the cerebellum [38]. Therefore, by synergistically combining the ecological potential of IVR with the network-level modulation of tACS, our protocol aims to provide the first mechanism-based rehabilitation for social prediction in cerebellar patients.

We hypothesize that the simultaneous application of IVR training and HD-tACS will yield synergistic effects, exceeding the benefits of single-modality interventions. Specifically, IVR provides the crucial experiential context: by ensuring a highly embodied experience and a strong sense of presence, it actively recruits the predictive coding mechanisms required to generate and update internal models of social interactions. Simultaneously, cerebellar HD-tACS modulates the underlying neural substrate to facilitate this relearning process. By entraining intrinsic neuronal oscillations, tACS aims to regulate the functional connectivity of cerebello-cerebral circuits, creating an optimal neurophysiological state for plasticity. Thus, we expect that tACS will potentiate the training effects induced by IVR, leading to a more robust restoration of social prediction abilities and better generalizability to daily life functioning.

A critical methodological advancement of this protocol is the implementation of a personalization strategy, fully aligning with the paradigm of precision medicine. First, regarding the behavioral training, we adopted a patient-centered approach to maximize motivation and compliance. The IVR content includes a set of diverse social scenarios and avatars that are specifically adapted to the patient’s age and everyday social scenarios. This ensures that the immersive experience is not only ecologically valid but also highly engaging. Complementing this behavioral tailoring, the neuromodulatory component is physiologically personalized. Building on evidence that tACS is most effective when matching endogenous rhythms (Arnold tongue principle) [74], we utilize EEG-guided IGF. Since gamma oscillations reflect the basal firing rate of Purkinje cells, locking the stimulation to the patient’s specific IGF ensures that the external current enhances rather than overrides intrinsic network dynamics. By tailoring both the immersive experience and the neurophysiological parameters, we aim to create a therapeutic environment that is uniquely adjusted to the individual needs of each patient.

Furthermore, the inclusion of both patients with CM and CA offers a unique opportunity to validate this multimodal approach across distinct neurophysiological substrates. While the differing developmental trajectories of these groups suggest divergent network organizations, a key objective is to evaluate whether these distinct etiologies differentially modulate the responsiveness to the proposed multimodal treatment. Specifically, in CM patients, the intervention interacts with neural circuits that have undergone atypical organization and compensatory rewiring since early life. In contrast, in CA patients, the treatment aims to counteract the degradation of previously established networks. By applying the same plasticity-inducing protocol (i.e., tACS + IVR) to these diverse pathological models, we can thus determine the extent to which the timing of the cerebellar damage—developmental versus acquired—influences the susceptibility to neuromodulation and the overall potential for functional restoration.

Importantly, this study addresses the critical issue of cerebellar tACS feasibility in pediatric populations. By adopting safety parameters tailored to adolescent anatomy (i.e., intensity limited to 1 mA), we aim to establish the tolerability of this technique in developing brains. Concurrently, to ensure the intervention’s applicability across varying levels of cognitive functioning, we recruit patients with moderate intellectual disability alongside those with mild or no impairment. This inclusive approach ensures the generalizability of results to a more representative sample of the cerebellar malformation population. Proving the viability of neuromodulation in this diverse group is a prerequisite for extending NIBS interventions to developmental disorders, potentially accelerating the adoption of cognitive rehabilitation across the lifespan.

On a broader scale, the present protocol embraces a transdiagnostic perspective that extends well beyond cerebellar disease. This approach is grounded in recent evidence suggesting that structural alterations within cerebellar circuitry are associated with a general liability for common mental disorders [75]. Consequently, validating a protocol that targets these specific circuits could identify therapeutic targets relevant for a wide range of conditions linked to cerebellar dysfunction, including neurodevelopmental (e.g., Autism Spectrum Disorder), neurological (e.g., Alzheimer’s disease, Parkinson’s disease), and psychiatric (e.g., Schizophrenia, mood disorders) conditions. In this light, the present protocol aims to lay the groundwork for the development of integrative interventions, translating cutting-edge findings in experimental neuroscience into rehabilitation procedures capable of offering long-lasting benefits in everyday life.

It is necessary, however, to acknowledge some limitations. First, a significant challenge stems from the anatomical heterogeneity inherent to the studied populations. In both malformative and degenerative patients, structural alterations are neither homogeneous nor precise, which may introduce variability in the neurophysiological response and limit anatomical standardization. In a related vein, although E-field modeling was performed to optimize the tACS montage, inter-individual anatomical variability (e.g., skull thickness, cerebellar geometry) might still influence the precise current distribution, a common constraint in non-invasive brain stimulation studies. Finally, regarding the IVR component, although severe ataxia is not a primary feature of our sample, the potential presence of mild vestibular or postural deficits in some patients could still pose a risk of cybersickness. To mitigate this, we have adopted specific safety measures, including seated administration of all sessions, frequent rest breaks, and constant monitoring of patient comfort.

In conclusion, the proposed protocol represents the first systematic attempt to translate the theoretical framework of cerebellar internal models into a concrete rehabilitation strategy. By synergistically targeting the neural and behavioral components of social prediction, this protocol aims not only to validate a novel therapeutic tool for cerebellar patients but also to provide crucial insights into the functional plasticity of the cerebellum. Ultimately, improving social prediction abilities holds the promise of restoring the behavioral fluidity necessary for successful social interactions, significantly impacting the quality of life of patients for whom specific treatments are often unavailable.

## Supporting information

S1 Checklist SPIRIT checklist of the study protocol. (DOCX)

S2 Protocol Protocol of the study (Italian and English versions). (ZIP)
