# Supplementary material for: Combining EEG-guided HD-tACS and Immersive Virtual Reality for Social Prediction Training in Congenital and Acquired Cerebellar Damage: A randomized sham-controlled trial protocol
Source: PLoS One. 2026 Aug 4;21(8):e0353955. doi: 10.1371/journal.pone.0353955 (PMC13436783; doi:10.1371/journal.pone.0353955)
Supplement: Supplementary file 2 [file pone.0353955.s002.zip › Protocol_ItalianVersion.pdf]

PRESENTAZIONE DELLO STUDIO

SINCRO: Spatiotemporal entrainment as Innovative Neuromodulation targeting Cerebello-cerebral circuits for enhancing Rehabilitation Outcomes of cognitive and social skills in progressive and acquired cerebellar diseases

(La sincronizzazione spazio-temporale come neuromodulazione innovativa mirata ai circuiti cerebello-cerebrali per migliorare i risultati della riabilitazione delle abilità cognitive e sociali nelle malattie cerebellari progressive e acquisite)

Responsabile dello Studio: Prof.ssa Maria Leggio

Ricercatori coinvolti: Prof. Marco Iosa, Prof. Francesco Di Russo, Dott.ssa Giusy Olivito, Dott.ssa Libera Siciliano, Dott. Andrea Casella, Dott.ssa Daniela De Bartolo (IRCCS Fondazione Santa Lucia),
Prof. Renato Borgatti, Prof.ssa Zaira Cattaneo, Dott. Andrea Ciricugno (IRCCS Fondazione Mondino)
Prof. Cosimo Urgesi (Università di Udine)

Durata del progetto: 3 anni
Finanziamento: Questo progetto è stato finanziato dal Ministero della Salute nell’ambito del programma di Ricerca Finalizzata.

Introduzione

Le alterazioni cerebellari risultano essere correlate non solo a deficit sensomotori, ma anche a una costellazione di disfunzioni cognitive, sociali e affettive, conosciute con il nome di sindrome cerebellare cognitivo-affettiva (CCAS) [1, 2], che colpisce bambini e adulti con atassia congenita o acquisita [3, 4]. Il cervelletto è visto come un nodo centrale nei circuiti neurali distribuiti responsabili delle funzioni sensomotorie, cognitive e affettive [5-7], dove opera principalmente generando simulazioni di eventi sotto forma di modelli interni [4, 8, 9]. Queste simulazioni si estendono a tutti i domini motori, cognitivi e sociali attraverso varie reti cerebello-cerebrali [5, 10, 11], giustificando il complesso quadro dei

sintomi della CCAS [1-4, 12, 13]. Nonostante queste conoscenze neuroscientifiche, esiste un bisogno insoddisfatto di riabilitazione della CCAS nei pazienti con alterazioni cerebellari. Prove preliminari provenienti dai nostri gruppi di ricerca hanno sostenuto l'efficacia di un allenamento basato sulla realtà virtuale (VR) per la cognizione sociale nei pazienti con atassia [14, 15]. Permettendo un'esperienza sensomotoria corporea di scenari complessi (sociali) in un ambiente altamente motivante e interattivo, la VR è uno strumento promettente per la riabilitazione [16]. La neuromodulazione della connettività cerebello-cerebrale può potenziare ulteriormente gli effetti del trattamento. La stimolazione cerebellare ha ricevuto un interesse crescente al fine di promuovere le funzioni motorie e non motorie e alleviare i sintomi della CCAS [17-23]. A questo scopo sembra particolarmente promettente la possibilità di personalizzare la neurostimolazione sulle dimensioni spaziale e temporale in funzione del modello individuale di dinamica dell'attività cerebrale mediante la stimolazione transcranica a corrente alternata (tACS) [24]. È stato dimostrato che la tACS sul cervelletto promuove la plasticità cerebellare e migliora le prestazioni motorie [25, 26]. La tACS è un metodo di neuromodulazione non invasivo che consente di modulare le oscillazioni cerebrali spontanee tramite correnti alternate a bassa intensità applicate sulla superficie del cuoio capelluto. La frequenza della stimolazione può essere sintonizzata sulla frequenza naturale delle oscillazioni cerebellari sottostanti, inducendo miglioramenti online e post-effetto delle prestazioni motorie [26, 27]. In effetti, varie frequenze sono state registrate in strati distinti del cervelletto: un ritmo oscillatorio di 50 Hz corrisponde alla frequenza di scarica basale delle cellule del Purkinje, mentre un ritmo di 6 Hz rientra nell'intervallo di oscillazioni coperto dalle cellule granulari [28, 29]. La tACS cerebellare a 50 Hz, tuttavia, si è dimostrata efficace nell'influenzare sia i potenziali evocati motori che le prestazioni motorie, mentre una stimolazione a 10 Hz è risultata non efficace [30]. Inoltre, l'utilizzo di un montaggio modificato ad anello centrale (noto come tACS ad alta definizione (HD) [31]) consente di guidare selettivamente le oscillazioni neurali nel circuito cerebellare.

Nessuno studio, tuttavia, ha affrontato gli effetti della tACS sulla riabilitazione cognitiva e socio-emotiva nell'atassia.

Scopo dello studio

L'obiettivo generale di questo progetto è quello di mettere a punto un protocollo di neuromodulazione per facilitare la neuroplasticità cerebello-cerebrale durante un training VR volto a potenziare la formazione di modelli interni di eventi fisici e sociali in pazienti

con malformazioni cerebellari congenite e disordini cerebellari neurodegenerativi. Ci aspettiamo che la stimolazione delle connessioni cerebello-cerebrali con la tACS, combinata con il training assistito da VR delle abilità di percezione sociale, migliori l'apprendimento implicito delle regolarità comportamentali di altri individui in pazienti con atassia progressiva. Questo potrebbe essere attivato anche in situazioni di vita quotidiana, con un reale miglioramento della vita sociale e affettiva dei pazienti e delle loro famiglie.

Obiettivi specifici dello studio.

O1. Mettere a punto un sistema immersivo di VR per valutare lo sviluppo di modelli predittivi nella percezione sociale e valutarne la fattibilità ed efficacia in (pre)-adolescenti, giovani adulti e adulti sani.

O2. Testare l'efficacia di una stimolazione cerebellare non invasiva a corrente alternata (tACS) in individui adulti sani attraverso la misurazione di parametri comportamentali ed elettroencefalografici (EEG)

O3. Validare l'efficacia di un approccio riabilitativo combinato basato sull'accoppiamento di un sistema di realtà virtuale con stimolazione cerebellare tACS in (pre)-adolescenti, giovani adulti e adulti con atassia congenita non progressiva o con disordini cerebellari neurodegenerativi

Nello specifico, gli esperimenti relativi all'obiettivo 1 su adulti sani, all'obiettivo 2 e all'obiettivo 3 su pazienti adulti con disordini cerebellari neurodegenerativi saranno svolti presso la Fondazione Santa Lucia (Responsabile Prof.ssa Maria Leggio) e sono descritti in dettaglio nelle sezioni seguenti.

Materiali e Metodi

Partecipanti

Per l'O1 si prevede di arruolare 1 gruppo di individui senza patologie neurologiche o psichiatriche per la creazione degli scenari di VR adatto per le fascia d'eta specifica N=24 adulti tra i 36 e i 60 anni.

Il gruppo di volontari sani (n=30) per l'O2 sarà arruolato sulla base dei seguenti criteri di inclusione: età compresa tra i 18 e i 35 anni. I criteri di esclusione saranno: condizioni psichiatriche e neurologiche attuali o pregresse, assunzione di farmaci che influenzano il sistema nervoso centrale e controindicazioni alla stimolazione tACS (presenza di impianti

metallici o elettronici nel cervello/cranio; presenza di dispositivi metallici o elettronici in altre parti del corpo; disturbi epilettici o episodi di perdita di coscienza o un grave trauma cranico; grave malattia cerebrale).

Per l'O3 verrà reclutato presso la Fondazione Santa Lucia 1 gruppo di 30 pazienti affetti da atrofia cerebellare degenerativa (età compresa tra i 18 e i 68 anni) . I criteri di inclusione saranno: più di 6 mesi di malattia; evidenza di diffusa atrofia cerebellare confermata tramite MRI 3T; QI >=75. I criteri di esclusione saranno: presenza di altre patologie neurologiche o psichiatriche; presenza di qualsiasi lesione extra-cerebellare alla MRI. l'International Cooperative Ataxia Rating Scale (ICARS) e la Cerebellar Cognitive Affective Scale (CCAS) saranno utilizzate per quantificare i sintomi cerebellari motori e cognitivi.

Procedura sperimentale e strumenti

Lo studio consta di tre livelli sperimentali:

- Sviluppo degli scenari di realtà virtuale validati in individui adulti sani
- Stimolazione mediante tACS, registrazioni EEG e compiti comportamentali in individui adulti sani
- Studio di tACS, EEG, compiti comportamentali e VR sui pazienti cerebellari adulti

Protocollo VR

L'allenamento in VR prevede che ogni partecipante effettui inizialmente 80 trial. In ogni trial il partecipante dovrà anticipare le mosse di un avatar virtuale facendo una scelta tra tre possibili. Ci saranno quattro possibili avatar, tre dei quali per l'80% delle volte faranno la stessa scelta, rendendo il loro comportamento prevedibile, mentre il quarto avatar farà scelte sempre diverse. Nel training con i pazienti le preferenze di ciascun avatar cambiano in ogni sessione giornaliera e, giorno dopo giorno, si prevede che i pazienti comprendano le preferenze degli avatar in quella sessione e ne anticipino il comportamento, aumentando così le capacità predittive. I contesti ambientali virtuali faranno riferimento ad un'ambientazione adatta alla fascia d'età (es. un ristorante e supermercato). L'interazione con il mondo virtuale avverrà attraverso un joystick. Alcuni avatar sceglieranno la stessa opzione nell'80% dei trial, altri agiranno in modo pseudo-random. Il partecipante per predire il comportamento dell'avatar dovrà capire quale sia la sua strategia. La

registrazione delle risposte del partecipante aiuterà a comprendere se questi ha sviluppato un modello del comportamento altrui.

Il sistema VR verrà generato in un ambiente compatibile con i visori a caschetto Oculus, già usati in altri protocolli approvati da questo comitato etico.

Stimolazione tACS

La stimolazione cerebellare non invasiva a corrente alternata (tACS) sarà applicata attraverso un montaggio ad alta definizione (HD-tACS 4x1), composto da cinque elettrodi circolari (circa 3 $cm^{2)}$. Le regioni target saranno gli emisferi cerebellari posteriori destro e sinistro, in linea con le evidenze che dimostrano che il contributo cerebellare alla cognizione sociale è sostenuto soprattutto dai settori cerebellari postero-laterali coinvolti nella rete di mentalizzazione dedicata alla comprensione degli stati mentali altrui, attraverso connessioni funzionali con le regioni di mentalizzazione corticali [5]. L'elettrodo attivo sarà posizionato sopra la regione cerebellare target, mentre quattro elettrodi di riferimento saranno posizionati in cerchio ed equidistanti dal centro (3 cm). La tACS verrà applicata alla frequenza gamma individuale (range: 40-60 Hz, rilevata tramite EEG), e consisterà in correnti sinusoidali (2 mA) applicate per 20 min, con una fase di salita/discesa di 30 secondi. Nella condizione di controllo (sham), saranno presenti solo le fasi di salita e discesa, ma per tutto il resto del tempo non sarà applicata corrente.

Registrazione EEG

l'EEG sarà registrato attraverso 64 elettrodi attivi montati sullo scalpo secondo il Sistema Internazionale 10-10 e con referenza agli elettrodi mastoidei. La frequenza di campionamento sarà di 250 Hz (filtro 0,01-80 Hz). I movimenti oculari saranno monitorati mediante elettro-oculogramma. Gli elettrodi saranno disinfettati prima di ogni utilizzo e il partecipante sarà disaccoppiato dalla rete elettrica grazie all’utilizzo di cavi a fibra ottica e degli amplificatori che funzionano con batterie da 3,6 V. Le registrazioni EEG saranno utilizzate a tre scopi:

1. Per individualizzare il trattamento prima di somministrare la tACS. A questo scopo si registrando cinque minuti di EEG spontaneo a occhi aperti e in stato di riposo. Questa registrazione permetterà di rilevare il picco di frequenza gamma individuale (IGF). L'IGF (intervallo: 40-60 Hz) è stato selezionato come frequenza target per la tACS in quanto particolarmente efficace per l'attività del cervelletto.

2. Per verificare l'effetto del trattamento (tACS) sui processi cerebrali sensoriali e cognitivi di base dei partecipanti. A questo scopo, prima e subito dopo il trattamento verranno acquisite ulteriori registrazioni EEG durante l'esecuzione di un compito visuo-motorio che permetterà anche di valutare funzioni comportamentali come il tempo di risposta e accuratezza. A livello EEG, saranno invece valutati i processi cerebrali sensoriali e cognitivi attraverso l'utilizzo della tecnica dei potenziali evento correlati (ERP) associati all'apparizione dallo stimolo nel compito visuo-motorio in entrambe le fasi sia pre e post-stimolo associate a funzioni cognitive bottom-up e top-down come l'attenzione e l'anticipazione del compito [32].
3. Eseguire analisi di coerenza parziale diretta (PDC) per studiare i cambiamenti della connettività funzionale cerebrale indotti dalla tACs e training VR durante lo stato di riposo e il compito visuo-motorio.

Consenso Informato

I partecipanti potranno accedere allo studio soltanto una volta che avranno firmato il consenso informato che include il consenso alla partecipazione e al trattamento dei dati. Vi sono diversi consensi informati specifici a seconda che il partecipante sarà un individuo sano che testerà solo il sistema VR, se sarà un soggetto sano che sarà valutato tramite EEG e che eseguirà il compito VR durante stimolazione tACS, o se sarà un paziente arruolato nello studio con tACS, EEG, compiti comportamentali e VR

Disegno sperimentale

Fase 1: Sviluppo di compiti virtuali e test su soggetti sani

Nella prima fase del protocollo verranno predisposti gli scenari e i task virtuali che verranno testati su individui adulti (36-60 anni, n=24). L'usabilità del sistema verrà valutata somministrando ai soggetti le scale validate USEQ e NASA-TLX. L'efficacia preliminare degli scenari sarà testata somministrando prima e dopo la sessione di realtà virtuale un compito di previsione degli stati mentali degli altri (intenzioni, emozioni o tratti di personalità) e un compito di controllo di previsione non sociale. Tutti i compiti hanno una struttura simile, con informazioni preliminari contestuali (sociali) offerte come stimoli priming, seguite da target ambigui, prevedibili o non prevedibili in base al contesto sociale. Anche se la decisione sul compito dipende dalle informazioni fornite dal target, dovrebbe essere influenzata dalla congruenza/incongruenza del prime contestuale (cioè, in linea con la previsione o in violazione della previsione).

Fase 2: Stimolazione tACS su soggetti sani

Verranno condotti quattro esperimenti, ognuno dei quali valuterà gli effetti della tACS sull'emisfero cerebellare destro e sinistro rispetto ad una condizione di controllo (sham). In ogni esperimento, durante la tACS, i partecipanti eseguiranno i compiti di priming contestuale nella previsione di stati mentali altrui e il compito di controllo di previsione non sociale come implementati nella fase 1. Verrà applicato un disegno sperimentale a misure ripetute, considerando la congruenza tra prime e target (congruenza vs. incongruenza), il tipo di stimolazione (tACS attiva a sinistra vs. tACS attiva a destra vs. sham) e la tempistica delle misurazioni EEG/ERP (prima vs. dopo la tACS) come fattori interni ai soggetti.

Fase 3: Studio di tACS, EEG, compiti comportamentali e VR *sui pazienti*

Trenta pazienti adulti (18-68 anni) con atrofia cerebellare degenerativa verranno arruolati presso la Fondazione Santa Lucia. I criteri di inclusione saranno: (i) più di 6 mesi di malattia, (ii) evidenza di atrofia cerebellare diffusa confermata tramite MRI 3T, (iii) QI >=75. I criteri di esclusione saranno: (i) presenza di altre condizioni neurologiche o psichiatriche e (ii) presenza di lesioni extracerebellari alla MRI. Verranno somministrate le scale ICARS (International Cooperative Ataxia Rating Scale) e la CCASS (Cerebellar Cognitive Affective/Schmahmann scale) per quantificare il danno motorio e cognitivo cerebellare.

In questa fase dello studio, i soggetti verranno trattati per due settimane con quattro sessioni a settimana di un’ora ciascuna, per un totale di otto sezioni. Sarà presente anche una seduta di follow-up dopo due mesi dalla fine dell’intervento. Immediatamente prima dell'inizio di ogni sessione di training, i pazienti saranno sottoposti a una sessione di tACS sul cervelletto. I pazienti saranno assegnati in modo randomizzato al gruppo sperimentale (stimolazione “real”) o al gruppo di controllo (stimolazione “sham”), utilizzando una lista di randomizzazione a blocchi generata elettronicamente da un computer.

Il risultato principale (primary outcome) sarà proprio la capacità di predire il comportamento altrui nei vari contesti attraverso misurazioni comportamentali e prevedere/anticipare gli eventi del compito visuo-motorio (parametri di accuratezza e tempo di risposta) e relativi correlati ERP. Poi come variabili secondarie saranno valutati i

punteggi a test psicologici (i.e. Vineland) e di percezione sociale (i.e., NEPSY-II) oltre ai già citati NASA-TLX e USEQ

Analisi statistiche

La dimensione del campione per ogni WP è stata calcolata con una power analysis a priori, utilizzando il software G-power (con l'opzione "as in SPSS") e stimando la dimensione dell'effetto dai dati preliminari rilevanti dei nostri gruppi di ricerca descritti di seguito

In un primo studio preliminare, un gruppo di pazienti con malformazioni cerebellari esegue un compito di previsione dell'azione di un modello che afferra un oggetto. utilizzando specifiche informazioni contestuali. I pazienti hanno mostrato deficit nell'utilizzo di informazioni contestuali precedenti per prevedere lo svolgimento delle azioni osservate (15). Deficit simili sono stati osservati per l'uso della modulazione contestuale per prevedere eventi fisici in un compito che manipolava l'associazione tra colore e forma di figure geometriche Altri risultati hanno anche mostrato una maggiore modulazione dell'informazione contestuale sulla predizione dell'azione dopo la tDCS catodica del cervelletto mediale in adulti sani (19).

La normalità della distribuzione dei dati sarà valutata utilizzando il test di Shapiro-Wilk, mentre il test di Levene sarà utilizzato per valutare l'omogeneità delle varianze. I test statistici parametrici saranno utilizzati se i dati risulteranno distribuiti normalmente, con le opportune correzioni se l'ipotesi della varianza non sarà soddisfatta. Per il trattamento dei dati verranno utilizzati modelli misti standard di Analisi della (Co)Varianza (AN(C)OVA). Per tutte le analisi è stata fissata una soglia di significatività di $p = 0.05$. I confronti post-hoc saranno eseguiti solo sui fattori statisticamente significativi per identificare la differenza significativa tra i livelli delle variabili indipendenti, applicando la correzione di Tukey per evitare l'inflazione dell'errore di primo tipo.

I dati sui partecipanti sani per l'O1 saranno inseriti in modelli misti ANOVA separati, uno per ogni compito, con la congruenza contestuale (prime contestuale congruente vs. incongruente) e la ripetizione (pre vs. post-training) come variabili a misura ripetuta. Sia le misure comportamentali (tempi di riposta e accuratezza) che quelle EEG/ERP, relative all'O2, saranno analizzate attraverso un'ANOVA a misure ripetute, separatamente per ciascun compito, con tACS (cerebellare destro attivo vs cerebellare sinistro attivo vs sham)

e congruenza contestuale (prime contestuale congruente vs incongruente) come variabili interne al soggetto.

Per lo studio relativo all'O3 saranno eseguiti confronti a-priori sulle caratteristiche demografiche e cliniche dei pazienti per confrontare il gruppo sperimentale con quello di controllo, al fine di controllare eventuali bias di campionamento. Per tenere conto delle differenze campionarie a-priori (anche se non statisticamente significative) verrà eseguita un'analisi di covarianza considerando la misura di outcome primario come variabile dipendente e come fattori fissi il trattamento (tACS attiva vs. sham come variabile tra i soggetti) e il tempo (pre, post-trattamento e follow-up) come variabile indipendente all'interno del soggetto. L'interazione dei suddetti fattori sarà considerata nel modello lineare generale. Considereremo anche l'età e il quoziente intellettivo dei partecipanti come possibili covariate. Se una di queste covariate risulterà statisticamente significativa, i dati verranno stratificati secondo valori omogenei della variabile significativa e verrà condotta un'analisi secondaria su sottogruppi omogenei per chiarire l'effetto delle covariate, correggendo il livello alfa per evitare la possibile inflazione dell'errore di primo tipo. In caso di dati non normalmente distribuiti, il primo approccio sarà l'adozione di tecniche di normalizzazione dei dati, come l'uso di punti z o punti percentuali; se questi tipi di normalizzazione falliranno, i dati saranno riassunti e analizzati utilizzando statistiche non parametriche. I dati saranno riportati in termini di valori mediani e quartili. Invece dell'analisi della varianza, verrà adottata l'analisi di Kruskal-Wallis per il confronto tra i soggetti e l'analisi di Friedman per il confronto dei parametri all'interno dei soggetti.

Risultati attesi

Per quanto riguarda la fase 1, ci aspettiamo che il training di realtà virtuale VR sia fattibile, coinvolgente e ben tollerato, come dimostrato dalle scale di usabilità e dai bassi tassi di abbandono. Il programma di training, infatti, comprenderà attività e scenari adattati all'età e al livello funzionale dei partecipanti. Ancora più importante, ci aspettiamo che la valutazione preliminare dell'efficacia del training di realtà virtuale si tradurrà in un miglioramento della capacità di anticipare le preferenze degli avatar. In particolare, ci aspettiamo che i partecipanti mostrino un apprendimento più rapido e accurato dell'associazione tra le alternative di scelte comportamentali e le intenzioni degli avatar, suggerendo una maggiore capacità nella costruzione di modelli interni delle preferenze comportamentali altrui. Questi effetti dovrebbero generalizzarsi ai compiti di priming contestuale nella previsione di stati mentali altrui, in cui i partecipanti dovrebbero fare più

affidamento sul prime contestuale per fornire le loro risposte. Analogamente, alla luce dei dati riportati in letteratura scientifica, ci aspettiamo che l'utilizzo della tACS cerebellare nelle fasi 2 e 3, rispetto alla stimolazione fittizia (sham), possa migliorare le prestazioni nei compiti di previsione sociale in adulti sani e in pazienti con alterazioni cerebellari, in particolare migliorando l'uso delle aspettative sociali per prevedere l'intenzione, le emozioni e i tratti di personalità degli altri. Si ipotizza che la tACS così strutturata, agendo su specifici bersagli cerebellari ed essendo personalizzata sulla specifica frequenza gamma, possa facilitare la capacità di formare modelli interni delle intenzioni altrui e potenziare gli effetti del training VR incentrato sulle abilità di percezione sociale in pazienti con disturbi cerebrali complessi che comportano alterazioni cerebellari. Si prevede che gli effetti si trasferiscano anche in compiti sensori-motori generici migliorando le capacità di anticipazione/preparazione al compito e attenzionali e alle attività della vita quotidiana e all'interazione sociale.

Il risultato finale di SINCRO sarà la proposta di un protocollo riabilitativo che combini il training VR e la stimolazione cerebrale non invasiva per alleviare i deficit di abilità sociali nei pazienti con disturbi legati alle funzioni cerebellari. La portabilità sia del training VR sia del dispositivo di stimolazione faciliterà la diffusione di questo protocollo nelle cliniche ospedaliere e in quelle ambulatoriali. Mentre i risultati forniranno prove specifiche di efficacia nei pazienti con alterazioni cerebellari, l'ampia fascia di età della popolazione testata in collaborazione con gli altri gruppi di ricerca favorirà lo sfruttamento dei risultati per estendere lo stesso protocollo in altri disturbi della cognizione sociale.

Bibliografia

1. Schmahmann JD. Disorders of the cerebellum: ataxia, dysmetria of thought, and the cerebellar cognitive affective syndrome. J Neuropsychiatry Clin Neurosci. 2004 Summer;16(3):367-78. doi: 10.1176/jnp.16.3.367. PMID: 15377747.
2. Argyropoulos GPD, van Dun K, Adamaszek M, Leggio M, Manto M, Masciullo M, Molinari M, Stoodley CJ, Van Overwalle F, Ivry RB, Schmahmann JD. The Cerebellar Cognitive Affective/Schmahmann Syndrome: a Task Force Paper. Cerebellum. 2020 Feb;19(1):102-125. doi: 10.1007/s12311-019-01068-8. PMID: 31522332; PMCID: PMC6978293.
3. Tavano A, Grasso R, Gagliardi C, Triulzi F, Bresolin N, Fabbro F, Borgatti R. Disorders of cognitive and affective development in cerebellar malformations. Brain. 2007 Oct;130(Pt 10):2646-60. doi: 10.1093/brain/awm201. Epub 2007 Sep 14. PMID: 17872929.
4. Tedesco AM, Chiricozzi FR, Clausi S, Lupo M, Molinari M, Leggio MG. The cerebellar cognitive profile. Brain. 2011 Dec;134(Pt 12):3672-86. doi: 10.1093/brain/awr266. Epub 2011 Oct 27. PMID: 22036960.
5. Van Overwalle F, Manto M, Cattaneo Z, Clausi S, Ferrari C, Gabrieli JDE, Guell X, Heleven E, Lupo M, Ma Q, Michelutti M, Olivito G, Pu M, Rice LC, Schmahmann JD, Siciliano L, Sokolov AA, Stoodley CJ, van Dun K, Vandervert L, Leggio M. Consensus Paper: Cerebellum and Social Cognition. Cerebellum. 2020 Dec;19(6):833-868. doi: 10.1007/s12311-020-01155-1. PMID: 32632709; PMCID: PMC7588399.
6. Baumann O, Borra RJ, Bower JM, Cullen KE, Habas C, Ivry RB, Leggio M, Mattingley JB, Molinari M, Moulton EA, Paulin MG, Pavlova MA, Schmahmann JD, Sokolov AA. Consensus paper: the role of the cerebellum in perceptual processes. Cerebellum. 2015 Apr;14(2):197-220. doi: 10.1007/s12311-014-0627-7. PMID: 25479821; PMCID: PMC4346664.
7. Mariën P, Ackermann H, Adamaszek M, Barwood CH, Beaton A, Desmond J, De Witte E, Fawcett AJ, Hertrich I, Küper M, Leggio M, Marvel C, Molinari M, Murdoch BE, Nicolson RI, Schmahmann JD, Stoodley CJ, Thürling M, Timmann D, Wouters E, Ziegler W. Consensus paper: Language and the cerebellum: an ongoing enigma. Cerebellum. 2014 Jun;13(3):386-410. doi: 10.1007/s12311-013-0540-5. PMID: 24318484; PMCID: PMC4090012.
8. Wolpert DM, Doya K, Kawato M. A unifying computational framework for motor control and social interaction. Philos Trans R Soc Lond B Biol Sci. 2003 Mar 29;358(1431):593-602. doi: 10.1098/rstb.2002.1238. PMID: 12689384; PMCID: PMC1693134.
9. Leggio MG, Chiricozzi FR, Clausi S, Tedesco AM, Molinari M. The neuropsychological profile of cerebellar damage: The sequencing hypothesis. Cortex. 2011 Jan;47(1):137-44. doi: 10.1016/j.cortex.2009.08.011. Epub 2009 Sep 6. PMID: 19786276.
10. Leggio M, Molinari M. Cerebellar sequencing: a trick for predicting the future. Cerebellum. 2015 Feb;14(1):35-8. doi: 10.1007/s12311-014-0616-x. PMID: 25331541.
11. Ito M. Control of mental activities by internal models in the cerebellum. Nat Rev Neurosci. 2008 Apr;9(4):304-13. doi: 10.1038/nrn2332. PMID: 18319727.
12. Lupo M, Siciliano L, Leggio M. From cerebellar alterations to mood disorders: A systematic review. Neurosci Biobehav Rev. 2019 Aug;103:21-28. doi:10.1016/j.neubiorev.2019.06.008. Epub 2019 Jun 10. PMID: 31195001.

13. Butti N, Corti C, Finisguerra A, Bardoni A, Borgatti R, Poggi G, Urgesi C. Cerebellar Damage Affects Contextual Priors for Action Prediction in Patients with Childhood Brain Tumor. Cerebellum. 2020 Dec;19(6):799-811. doi: 10.1007/s12311-020-01168-w. PMID: 32699945.
14. Butti N, Biffi E, Genova C, Romaniello R, Redaelli DF, Reni G, Borgatti R, Urgesi C. Virtual Reality Social Prediction Improvement and Rehabilitation Intensive Training (VR-SPIRIT) for paediatric patients with congenital cerebellar diseases: study protocol of a randomised controlled trial. Trials. 2020 Jan 14;21(1):82. doi: 10.1186/s13063-019-4001-4. PMID: 31937355; PMCID: PMC6961250.
15. Urgesi C, Butti N, Finisguerra A, Biffi E, Valente EM, Romaniello R, Borgatti R. Social prediction in pediatric patients with congenital, non-progressive malformations of the cerebellum: From deficits in predicting movements to rehabilitation in virtual reality. Cortex. 2021 Nov;144:82-98. doi: 10.1016/j.cortex.2021.08.008. Epub 2021 Sep 24. PMID: 34662720.
16. Tieri G, Morone G, Paolucci S, Iosa M. Virtual reality in cognitive and motor rehabilitation: facts, fiction and fallacies. Expert Rev Med Devices. 2018 Feb;15(2):107-117. doi: 10.1080/17434440.2018.1425613. Epub 2018 Jan 10. PMID: 29313388.
17. Miterko LN, Baker KB, Beckinghausen J, Bradnam LV, Cheng MY, Cooperrider J, DeLong MR, Gornati SV, Hallett M, Heck DH, Hoebeek FE, Kouzani AZ, Kuo SH, Louis ED, Machado A, Manto M, McCambridge AB, Nitsche MA, Taib NOB, Popa T, Tanaka M, Timmann D, Steinberg GK, Wang EH, Wichmann T, Xie T, Sillitoe RV. Consensus Paper: Experimental Neurostimulation of the Cerebellum. Cerebellum. 2019 Dec;18(6):1064-1097. doi: 10.1007/s12311-019-01041-5. PMID: 31165428; PMCID: PMC6867990.
18. Cattaneo Z, Ferrari C, Ciricugno A, Heleven E, Schutter DJLG, Manto M, Van Overwalle F. New Horizons on Non-invasive Brain Stimulation of the Social and Affective Cerebellum. Cerebellum. 2022 Jun;21(3):482-496. doi: 10.1007/s12311-021-01300-4. Epub 2021 Jul 16. PMID: 34270081.
19. Oldrati V, Ferrari E, Butti N, Cattaneo Z, Borgatti R, Urgesi C, Finisguerra A. How social is the cerebellum? Exploring the effects of cerebellar transcranial direct current stimulation on the prediction of social and physical events. Brain Struct Funct. 2021 Apr;226(3):671-684. doi: 10.1007/s00429-020-02198-0. Epub 2021 Jan 11. PMID: 33426567.
20. Ferrari C, Ciricugno A, Urgesi C, Cattaneo Z. Cerebellar contribution to emotional body language perception: a TMS study. Soc Cogn Affect Neurosci. 2019 Oct 7;17(1):81–90. doi: 10.1093/scan/nsz074. Epub ahead of print. PMID: 31588511; PMCID: PMC8824541.
21. Ciricugno A, Ferrari C, Rusconi ML, Cattaneo Z. The left posterior cerebellum is involved in orienting attention along the mental number line: An online-TMS study. Neuropsychologia. 2020 Jun;143:107497. doi: 10.1016/j.neuropsychologia.2020.107497. Epub 2020 May 12. PMID: 32413432.
22. Ferrari C, Ciricugno A, Battelli L, Grossman ED, Cattaneo Z. Distinct Cerebellar regions for Body Motion Discrimination. Soc Cogn Affect Neurosci. 2019 Dec 10;17(1):72–80. doi: 10.1093/scan/nsz088. Epub ahead of print. PMID: 31820788; PMCID: PMC8824544.
23. Oldrati V, Schutter DJLG. Targeting the Human Cerebellum with Transcranial Direct Current Stimulation to Modulate Behavior: a Meta-Analysis. Cerebellum. 2018 Apr;17(2):228-236. doi: 10.1007/s12311-017-0877-2. PMID: 28786014; PMCID: PMC5849643.

24. Thut G, Bergmann TO, Fröhlich F, Soekadar SR, Brittain JS, Valero-Cabré A, Sack AT, Miniussi C, Antal A, Siebner HR, Ziemann U, Herrmann CS. Guiding transcranial brain stimulation by EEG/MEG to interact with ongoing brain activity and associated functions: A position paper. Clin Neurophysiol. 2017 May;128(5):843-857. doi: 10.1016/j.clinph.2017.01.003. Epub 2017 Jan 29. PMID: 28233641; PMCID: PMC5385293.
25. Miyaguchi S, Otsuru N, Kojima S, Yokota H, Saito K, Inukai Y, Onishi H. The effect of gamma tACS over the M1 region and cerebellar hemisphere does not depend on current intensity. J Clin Neurosci. 2019 Jul;65:54-58. doi: 10.1016/j.jocn.2019.03.045. Epub 2019 Apr 3. PMID: 30954355.
26. Naro A, Leo A, Russo M, Cannavò A, Milardi D, Bramanti P, Calabrò RS. Does Transcranial Alternating Current Stimulation Induce Cerebellum Plasticity? Feasibility, Safety and Efficacy of a Novel Electrophysiological Approach. Brain Stimul. 2016 May-Jun;9(3):388-395. doi: 10.1016/j.brs.2016.02.005. Epub 2016 Feb 12. PMID: 26946958.
27. Naro A, Bramanti A, Leo A, Manuli A, Sciarrone F, Russo M, Bramanti P, Calabrò RS. Effects of cerebellar transcranial alternating current stimulation on motor cortex excitability and motor function. Brain Struct Funct. 2017 Aug;222(6):2891-2906. doi: 10.1007/s00429-016-1355-1. Epub 2017 Jan 7. PMID: 28064346.
28. De Zeeuw CI, Hoebeek FE, Schonewille M. Causes and consequences of oscillations in the cerebellar cortex. Neuron. 2008 Jun 12;58(5):655-8. doi: 10.1016/j.neuron.2008.05.019. PMID: 18549777.
29. D'Angelo E, Nieus T, Maffei A, Armano S, Rossi P, Taglietti V, Fontana A, Naldi G. Theta-frequency bursting and resonance in cerebellar granule cells: experimental evidence and modeling of a slow k+-dependent mechanism. J Neurosci. 2001 Feb 1;21(3):759-70. doi: 10.1523/JNEUROSCI.21-03-00759.2001. PMID: 11157062; PMCID: PMC6762330.
30. Naro A, Milardi D, Cacciola A, Russo M, Sciarrone F, La Rosa G, Bramanti A, Bramanti P, Calabrò RS. What Do We Know About the Influence of the Cerebellum on Walking Ability? Promising Findings from Transcranial Alternating Current Stimulation. Cerebellum. 2017 Aug;16(4):859-867. doi: 10.1007/s12311-017-0859-4. PMID: 28456901.
31. Heise KF, Kortzorg N, Saturnino GB, Fujiyama H, Cuypers K, Thielscher A, Swinnen SP. Evaluation of a Modified High-Definition Electrode Montage for Transcranial Alternating Current Stimulation (tACS) of Pre-Central Areas. Brain Stimul. 2016 Sep-Oct;9(5):700-704. doi: 10.1016/j.brs.2016.04.009. Epub 2016 Apr 14. PMID: 27160465.
32. Di Russo F, M B, V B, Rl P, S P, F Q, D S. Normative event-related potentials from sensory and cognitive tasks reveal occipital and frontal activities prior and following visual events. Neuroimage. 2019 Aug 1;196:173-187. doi: 10.1016/j.neuroimage.2019.04.033. Epub 2019 Apr 11. PMID: 30981857.
