# Supplementary material for: Combining EEG-guided HD-tACS and Immersive Virtual Reality for Social Prediction Training in Congenital and Acquired Cerebellar Damage: A randomized sham-controlled trial protocol
Source: PLoS One. 2026 Aug 4;21(8):e0353955. doi: 10.1371/journal.pone.0353955 (PMC13436783; doi:10.1371/journal.pone.0353955)
Supplement: Supplementary file 2 [file pone.0353955.s002.zip › Protocol_EnglishVersion.pdf]

SINCRO: Spatiotemporal entrainment as Innovative Neuromodulation targeting Cerebello-cerebral circuits for enhancing Rehabilitation Outcomes of cognitive and social skills in progressive and acquired cerebellar diseases

Principal Investigator: Prof.ssa Maria Leggio

Research Team: Prof. Marco Iosa, Prof. Francesco Di Russo, Dott.ssa Giusy Olivito, Dott.ssa Libera Siciliano, Dott. Andrea Casella, Dott.ssa Daniela De Bartolo (IRCCS Santa Lucia),
Prof. Renato Borgatti, Prof.ssa Zaira Cattaneo, Dott. Andrea Ciricugno (IRCCS Mondino Foundation)
Prof. Cosimo Urgesi (University of Udine)

Project duration: 3 years
Funding: This work was supported by Italian Ministry of Health (RF-2021-12374279).

Introduction

Cerebellar alterations are correlated not only with sensorimotor deficits but also with a constellation of cognitive, social, and affective dysfunctions, known as Cerebellar Cognitive Affective Syndrome (CCAS) [1, 2], which affects both children and adults with congenital or acquired ataxia [3, 4]. The cerebellum is viewed as a central node in distributed neural circuits responsible for sensorimotor, cognitive, and affective functions [5-7], where it operates primarily by generating simulations of events in the form of internal models [4, 8, 9]. These simulations extend to all motor, cognitive, and social domains through various cerebello-cerebral networks [5, 10, 11], accounting for the complex symptomatic profile of CCAS [1-4, 12, 13].

Despite these neuroscientific insights, there remains an unmet need for CCAS rehabilitation in patients with cerebellar alterations. Preliminary evidence from our research groups has supported the efficacy of Virtual Reality (VR)-based training for social cognition in ataxia patients [14, 15]. By providing a bodily sensorimotor experience of complex (social) scenarios within a highly motivating and interactive environment, VR represents a promising tool for rehabilitation [16].

Neuromodulation of cerebello-cerebral connectivity may further enhance treatment effects. Cerebellar stimulation has received increasing interest as a means to promote motor and

non-motor functions and alleviate CCAS symptoms [17-23]. To this end, the possibility of personalizing neurostimulation across spatial and temporal dimensions—based on individual brain activity dynamics through transcranial Alternating Current Stimulation (tACS)—appears particularly promising [24].

Cerebellar tACS has been shown to promote cerebellar plasticity and improve motor performance [25, 26]. tACS is a non-invasive neuromodulation method that allows for the modulation of spontaneous brain oscillations via low-intensity alternating currents applied to the scalp surface. The stimulation frequency can be tuned to the natural frequency of the underlying cerebellar oscillations, inducing online and post-effect improvements in motor performance [26, 27]. Indeed, various frequencies have been recorded in distinct cerebellar layers: a 50 Hz oscillatory rhythm corresponds to the basal firing rate of Purkinje cells, while a 6 Hz rhythm falls within the range of oscillations covered by granule cells [28, 29]. However, cerebellar tACS at 50 Hz has proven effective in influencing both motor-evoked potentials and motor performance, whereas 10 Hz stimulation was found to be ineffective [30]. Furthermore, the use of a modified center-ring montage (known as High-Definition (HD)-tACS [31]) allows for the selective entrainment of neural oscillations within the cerebellar circuit. To date, however, no study has addressed the effects of tACS on cognitive and socio-emotional rehabilitation in ataxia.

Study aim

The overall objective of this project is to develop a neuromodulation protocol to facilitate cerebello-cerebral neuroplasticity during Virtual Reality (VR) training, aimed at enhancing the formation of internal models of physical and social events in patients with congenital cerebellar malformations and neurodegenerative cerebellar disorders. We hypothesize that stimulating cerebello-cerebral connections using tACS, combined with VR-assisted training of social perception skills, will improve the implicit learning of behavioral regularities of other individuals in patients with progressive ataxia. This may also be translated to daily life situations, leading to a tangible improvement in the social and affective lives of both patients and their families.

Specific objectives

O1. To develop an immersive VR system for evaluating the development of predictive models in social perception, and to assess its feasibility and efficacy in (pre-)adolescents, young adults, and healthy adults.

O2. To test the efficacy of non-invasive cerebellar transcranial Alternating Current Stimulation (tACS) in healthy adult individuals by measuring behavioral and electroencephalographic (EEG) parameters.

O3. To validate the efficacy of a combined rehabilitative approach based on the pairing of a Virtual Reality system with cerebellar tACS in (pre-)adolescents, young adults, and adults with non-progressive congenital malformation or neurodegenerative cerebellar disorders.

Methods

Participants

For O1, a group of individuals without neurological or psychiatric pathologies will be enrolled to develop VR scenarios suitable for the specific age group (N=24 adults, aged 36 to 60 years).

The group of healthy volunteers (n=30) for O2 will be enrolled based on the following inclusion criteria: age between 18 and 35 years. Exclusion criteria will include: current or previous psychiatric and neurological conditions; use of medications affecting the central nervous system; and contraindications to tACS stimulation (presence of metallic or electronic implants in the brain/skull; presence of metallic or electronic devices in other parts of the body; seizure disorders, episodes of loss of consciousness, or severe traumatic brain injury; and severe brain disease).

For O3, a group of 30 patients with congenital cerebellar malformation (CM) (age > 12 years old) will be recruited at IRCCS Mondino Foundation, and a group of 30 patients with degenerative cerebellar atrophy (CA) (aged 18 to 68 years) will be recruited at the Fondazione Santa Lucia. Inclusion criteria for CM patients include: documented malformations confined to the cerebellum confirmed by 3T brain MRI; age ranging from 12 to 32 years; IQ >=50; and absence of extra-cerebellar malformations on conventional brain MRI. Inclusion criteria for CA patients include: evidence of diffuse cerebellar atrophy; disease duration of more than 6 months; IQ >=75; and absence of any cortical lesion on conventional brain MRI. Exclusion criteria for both groups include: presence of severe motor and visual impairments, as well as neurodevelopmental (e.g., autism), neurological, or psychiatric disorders that could interfere with task execution and protocol

compliance; and presence of any contraindication for tACS. The ICARS (International Cooperative Ataxia Rating Scale) and the CCAS (Cerebellar Cognitive Affective/Schmahmann Scale) will be administered to quantify cerebellar motor and cognitive impairment.

Experimental design

The study consists of three experimental phases:

- Development of Virtual Reality scenarios validated in healthy adult individuals.
- tACS stimulation, EEG recordings, and behavioral tasks in healthy adult individuals.
- Study of tACS, EEG, behavioral tasks, and VR in cerebellar patients.

VR training protocol

The VR training consists of an initial session of 80 trials per participant. In each trial, the participant must anticipate the moves of a virtual avatar by choosing from three possible options. There will be four different avatars: three of them will make the same choice 80% of the time, making their behavior predictable, while the fourth avatar will make consistently varying choices. In the patient training, each avatar's preferences change in every daily session. Over the course of the training, patients are expected to identify the avatars' specific preferences for that session and anticipate their behavior, thereby increasing their predictive capabilities. The virtual environments will feature age-appropriate settings (e.g., a restaurant or a supermarket). Interaction with the virtual world will be managed via a joystick. While some avatars will select the same option in 80% of the trials, others will act in a pseudo-random manner. To predict the avatar's behavior, the participant must deduce its underlying strategy. The recording of participant responses will help determine whether they have developed an internal model of others' behavior.

The VR system will be developed in an environment compatible with Oculus head-mounted displays (HMDs), which have been previously utilized in other protocols approved by this Ethics Committee.

tACS

Non-invasive cerebellar transcranial Alternating Current Stimulation (tACS) will be delivered via a High-Definition montage (4x1 HD-tACS), consisting of five circular electrodes (approximately 3cm^2 each). The target regions will be the right and left posterior cerebellar hemispheres, consistent with evidence showing that the cerebellar contribution to social cognition is primarily supported by the postero-lateral cerebellar sectors. These sectors are involved in the mentalizing network dedicated to understanding others' mental states through functional connections with cortical mentalizing regions [5].The active electrode will be positioned over the target cerebellar region, while four reference electrodes will be arranged in a circle, equidistant (3 cm) from the center. The tACS will be applied at the individual gamma frequency (range: 40–60 Hz, as detected via EEG) and will consist of sinusoidal currents (2 mA) applied for 20 minutes, including a 30-second ramp-up/ramp-down phase. In the control (sham) condition, only the ramp-up and ramp-down phases will be present, with no current applied for the remainder of the session.

EEG recording

EEG will be recorded using 64 active scalp electrodes positioned according to the International 10-10 System, with reference to the mastoid electrodes. The sampling rate will be 250 Hz (filter: 0.01–80 Hz). Eye movements will be monitored via electro-oculography (EOG). Electrodes will be disinfected before each use, and the participant will be decoupled from the electrical grid through the use of fiber-optic cables and amplifiers powered by 3.6 V batteries. EEG recordings will serve three primary purposes:

1. Individualization of tACS Treatment: Prior to stimulation, five minutes of resting-state EEG (eyes open) will be recorded to detect the Individual Gamma Frequency (IGF) peak. The IGF (range: 40–60 Hz) has been selected as the target frequency for tACS, as it is particularly effective for modulating cerebellar activity.
2. Assessment of Treatment Effects: To verify the impact of tACS on the participants' basic sensory and cognitive brain processes, additional EEG recordings will be acquired before and immediately after the treatment during a visuomotor task. This task will also allow for the evaluation of behavioral functions such as response time and accuracy. At the electrophysiological level, sensory and cognitive processes will be assessed using the Event-Related Potential (ERP) technique associated with stimulus onset. Both pre- and post-stimulus phases will be analyzed to investigate

bottom-up and top-down cognitive functions, such as attention and task anticipation [32].

3. Connectivity Analysis: Partial Directed Coherence (PDC) analysis will be performed to study changes in functional brain connectivity induced by tACS and VR training during both resting-state and the visuomotor task.

Informed consent

Participants may only access the study once they have signed the informed consent form, which includes consent for participation and data processing. There are several specific informed consent forms depending on whether the participant is a healthy individual testing only the VR system, a healthy subject undergoing EEG evaluation and the VR task during tACS stimulation, or a patient enrolled in the study involving tACS, EEG, behavioral tasks, and VR. For minor patients, informed consent will also be required from parents or legal guardians.

Experimental design

Phase 1: Development of virtual tasks and testing on healthy subjects

In the first phase of the protocol, virtual scenarios and tasks will be developed and tested on adult individuals (36-60 years, n=24). System usability will be assessed by administering the validated USEQ and NASA-TLX scales. Preliminary efficacy of the scenarios will be tested by administering, before and after the VR session, a task for predicting others' mental states (intentions, emotions, or personality traits) and a non-social prediction control task. All tasks share a similar structure, featuring preliminary contextual (social) information provided as priming stimuli, followed by ambiguous targets that are either predictable or unpredictable based on the social context. Although the task decision depends on the information provided by the target, it should be influenced by the congruence/incongruence of the contextual prime (i.e., in line with the prediction or in violation of it).

Phase 2: tACS stimulation on healthy subjects

Four experiments will be conducted, each evaluating the effects of tACS on the right and left cerebellar hemispheres compared to a control (sham) condition. In each experiment, during tACS, participants will perform the contextual priming tasks for predicting others'

mental states and the non-social prediction control task as implemented in Phase 1. A repeated-measures experimental design will be applied, considering prime-target congruence (congruence vs. incongruence), stimulation type (left active tACS vs. right active tACS vs. sham), and the timing of EEG/ERP measurements (before vs. after tACS) as within-subject factors.

Phase 3: Study of tACS, EEG, behavioral tasks, and VR in patients

Thirty patients with degenerative cerebellar atrophy (CA) will be recruited at the Fondazione Santa Lucia, and 30 patients with congenital cerebellar malformations (CM) will be recruited at the IRCCS Mondino. Inclusion criteria for CM patients include: documented malformations confined to the cerebellum confirmed by 3T brain MRI; age ranging from 12 to 32 years; IQ >=50; and absence of extra-cerebellar malformations on conventional brain MRI. Inclusion criteria for CA patients include: evidence of diffuse cerebellar atrophy; disease duration of more than 6 months; IQ >=75; and absence of any cortical lesion on conventional brain MRI. Exclusion criteria for both groups include: presence of severe motor and visual impairments, as well as neurodevelopmental (e.g., autism), neurological, or psychiatric disorders that could interfere with task execution and protocol compliance; and presence of any contraindication for tACS. The ICARS (International Cooperative Ataxia Rating Scale) and the CCAS (Cerebellar Cognitive Affective/Schmahmann Scale) will be administered to quantify cerebellar motor and cognitive impairment.

In this phase of the study, subjects will be treated for two weeks with four one-hour sessions per week, for a total of eight sessions. A follow-up session will also take place two months after the end of the intervention. Immediately before the start of each training session, patients will undergo a cerebellar tACS session. Patients will be randomly assigned to either the experimental group ("real" stimulation) or the control group ("sham" stimulation) using a computer-generated block randomization list.

The primary outcome will be the ability to predict others' behavior across various contexts through behavioral measurements and the ability to predict/anticipate events in the visuomotor task (accuracy and response time parameters) and related ERP correlates. Secondary variables will include scores from psychological tests (e.g., Vineland) and social perception tests (e.g., NEPSY-II), in addition to the previously mentioned NASA-TLX and USEQ.

Statistical analyses

The sample size for each WP was calculated using an a priori power analysis with G*Power software (using the "as in SPSS" option), estimating the effect size from relevant preliminary data from our research groups described below. In an initial preliminary study, a group of patients with cerebellar malformations performed a task predicting the action of a model grasping an object using specific contextual information. Patients showed deficits in using prior contextual information to predict the unfolding of observed actions [15]. Similar deficits were observed in the use of contextual modulation to predict physical events in a task manipulating the association between the color and shape of geometric figures. Other results also showed increased modulation of contextual information on action prediction following cathodal tDCS of the medial cerebellum in healthy adults [19]. Data distribution normality will be assessed using the Shapiro-Wilk test, while Levene's test will be used to evaluate the homogeneity of variances. Parametric statistical tests will be used if data are normally distributed, with appropriate corrections applied if the variance hypothesis is not met. Standard mixed-model Analysis of (Co)Variance (AN(C)OVA) will be used for data processing. For all analyses, the significance threshold is set at $p = 0.05$. Post-hoc comparisons will be performed only on statistically significant factors to identify significant differences between the levels of independent variables, applying the Tukey correction to avoid Type I error inflation.

Data from healthy participants for O1 will be entered into separate mixed-model ANOVAs, one for each task, with contextual congruence (congruent vs. incongruent contextual prime) and repetition (pre- vs. post-training) as repeated-measures variables.

For O2, both behavioral measures (response times and accuracy) and EEG/ERP measures will be analyzed via repeated-measures ANOVA, separately for each task, with tACS (active right cerebellar vs. active left cerebellar vs. sham) and contextual congruence (congruent vs. incongruent contextual prime) as within-subject variables.

For the study related to O3, a priori comparisons of patients' demographic and clinical characteristics will be performed to compare the experimental and control groups and control for potential sampling bias. To account for a priori sample differences (even if not statistically significant), an analysis of covariance will be conducted, considering the primary outcome measure as the dependent variable, treatment (active tACS vs. sham) as the between-subjects fixed factor, and time (pre-, post-treatment, and follow-up) as the within-subject independent variable. The interaction of these factors will be considered in

the general linear model. Participants' age and IQ will also be considered as possible covariates. If any of these covariates prove statistically significant, data will be stratified according to homogeneous values of the significant variable, and a secondary analysis on homogeneous subgroups will be conducted to clarify the covariate effect, adjusting the alpha level to prevent Type I error inflation. In case of non-normally distributed data, the first approach will involve data normalization techniques, such as the use of z-scores or percentiles; if these normalization methods fail, data will be summarized and analyzed using non-parametric statistics. Data will be reported as medians and quartiles. Instead of ANOVA, the Kruskal-Wallis analysis will be adopted for between-subjects comparisons and the Friedman analysis for within-subjects parameter comparisons.

Expected results

Regarding Phase 1, we expect the VR training to be feasible, engaging, and well-tolerated, as demonstrated by usability scales and low dropout rates. Indeed, the training program will include activities and scenarios tailored to the participants' age and functional level. More importantly, we anticipate that the preliminary efficacy assessment of the VR training will translate into an improved ability to anticipate avatar preferences. Specifically, we expect participants to show faster and more accurate learning of the association between behavioral choice alternatives and avatar intentions, suggesting an enhanced capacity to construct internal models of others' behavioral preferences. These effects should generalize to contextual priming tasks in predicting others' mental states, where participants are expected to rely more heavily on the contextual prime to provide their responses.

Similarly, in light of data reported in the scientific literature, we expect that the use of cerebellar tACS in Phases 2 and 3, compared to sham stimulation, will improve performance in social prediction tasks in both healthy adults and patients with cerebellar alterations—specifically by enhancing the use of social expectations to predict others' intentions, emotions, and personality traits. It is hypothesized that tACS structured in this manner, by acting on specific cerebellar targets and being personalized to the specific gamma frequency, can facilitate the ability to form internal models of others' intentions and bolster the effects of VR training focused on social perception skills in patients with complex brain disorders involving cerebellar alterations. These effects are also expected to transfer to general sensorimotor tasks, improving task anticipation/preparation and attentional skills, as well as activities of daily living and social interaction.

The final outcome of SINCRO will be the proposal of a rehabilitative protocol that combines VR training and non-invasive brain stimulation to alleviate social skill deficits in patients with disorders related to cerebellar functions. The portability of both the VR training and the stimulation device will facilitate the dissemination of this protocol in hospital and outpatient clinics. While the results will provide specific evidence of efficacy in patients with cerebellar alterations, the wide age range of the population tested in collaboration with other research groups will favor the exploitation of these findings to extend the same protocol to other social cognition disorders.

References

1. Schmahmann JD. Disorders of the cerebellum: ataxia, dysmetria of thought, and the cerebellar cognitive affective syndrome. J Neuropsychiatry Clin Neurosci. 2004 Summer;16(3):367-78. doi: 10.1176/jnp.16.3.367. PMID: 15377747.
2. Argyropoulos GPD, van Dun K, Adamaszek M, Leggio M, Manto M, Masciullo M, Molinari M, Stoodley CJ, Van Overwalle F, Ivry RB, Schmahmann JD. The Cerebellar Cognitive Affective/Schmahmann Syndrome: a Task Force Paper. Cerebellum. 2020 Feb;19(1):102-125. doi: 10.1007/s12311-019-01068-8. PMID: 31522332; PMCID: PMC6978293.
3. Tavano A, Grasso R, Gagliardi C, Triulzi F, Bresolin N, Fabbro F, Borgatti R. Disorders of cognitive and affective development in cerebellar malformations. Brain. 2007 Oct;130(Pt 10):2646-60. doi: 10.1093/brain/awm201. Epub 2007 Sep 14. PMID: 17872929.
4. Tedesco AM, Chiricozzi FR, Clausi S, Lupo M, Molinari M, Leggio MG. The cerebellar cognitive profile. Brain. 2011 Dec;134(Pt 12):3672-86. doi: 10.1093/brain/awr266. Epub 2011 Oct 27. PMID: 22036960.
5. Van Overwalle F, Manto M, Cattaneo Z, Clausi S, Ferrari C, Gabrieli JDE, Guell X, Heleven E, Lupo M, Ma Q, Michelutti M, Olivito G, Pu M, Rice LC, Schmahmann JD, Siciliano L, Sokolov AA, Stoodley CJ, van Dun K, Vandervert L, Leggio M. Consensus Paper: Cerebellum and Social Cognition. Cerebellum. 2020 Dec;19(6):833-868. doi: 10.1007/s12311-020-01155-1. PMID: 32632709; PMCID: PMC7588399.
6. Baumann O, Borra RJ, Bower JM, Cullen KE, Habas C, Ivry RB, Leggio M, Mattingley JB, Molinari M, Moulton EA, Paulin MG, Pavlova MA, Schmahmann JD, Sokolov AA. Consensus paper: the role of the cerebellum in perceptual processes. Cerebellum. 2015 Apr;14(2):197-220. doi: 10.1007/s12311-014-0627-7. PMID: 25479821; PMCID: PMC4346664.
7. Mariën P, Ackermann H, Adamaszek M, Barwood CH, Beaton A, Desmond J, De Witte E, Fawcett AJ, Hertrich I, Küper M, Leggio M, Marvel C, Molinari M, Murdoch BE, Nicolson RI, Schmahmann JD, Stoodley CJ, Thürling M, Timmann D, Wouters E, Ziegler W. Consensus paper: Language and the cerebellum: an ongoing enigma. Cerebellum. 2014 Jun;13(3):386-410. doi: 10.1007/s12311-013-0540-5. PMID: 24318484; PMCID: PMC4090012.
8. Wolpert DM, Doya K, Kawato M. A unifying computational framework for motor control and social interaction. Philos Trans R Soc Lond B Biol Sci. 2003 Mar 29;358(1431):593-602. doi: 10.1098/rstb.2002.1238. PMID: 12689384; PMCID: PMC1693134.
9. Leggio MG, Chiricozzi FR, Clausi S, Tedesco AM, Molinari M. The neuropsychological profile of cerebellar damage: The sequencing hypothesis. Cortex. 2011 Jan;47(1):137-44. doi: 10.1016/j.cortex.2009.08.011. Epub 2009 Sep 6. PMID: 19786276.
10. Leggio M, Molinari M. Cerebellar sequencing: a trick for predicting the future. Cerebellum. 2015 Feb;14(1):35-8. doi: 10.1007/s12311-014-0616-x. PMID: 25331541.
11. Ito M. Control of mental activities by internal models in the cerebellum. Nat Rev Neurosci. 2008 Apr;9(4):304-13. doi: 10.1038/nrn2332. PMID: 18319727.
12. Lupo M, Siciliano L, Leggio M. From cerebellar alterations to mood disorders: A systematic review. Neurosci Biobehav Rev. 2019 Aug;103:21-28. doi:10.1016/j.neubiorev.2019.06.008. Epub 2019 Jun 10. PMID: 31195001.

13. Butti N, Corti C, Finisguerra A, Bardoni A, Borgatti R, Poggi G, Urgesi C. Cerebellar Damage Affects Contextual Priors for Action Prediction in Patients with Childhood Brain Tumor. Cerebellum. 2020 Dec;19(6):799-811. doi: 10.1007/s12311-020-01168-w. PMID: 32699945.
14. Butti N, Biffi E, Genova C, Romaniello R, Redaelli DF, Reni G, Borgatti R, Urgesi C. Virtual Reality Social Prediction Improvement and Rehabilitation Intensive Training (VR-SPIRIT) for paediatric patients with congenital cerebellar diseases: study protocol of a randomised controlled trial. Trials. 2020 Jan 14;21(1):82. doi: 10.1186/s13063-019-4001-4. PMID: 31937355; PMCID: PMC6961250.
15. Urgesi C, Butti N, Finisguerra A, Biffi E, Valente EM, Romaniello R, Borgatti R. Social prediction in pediatric patients with congenital, non-progressive malformations of the cerebellum: From deficits in predicting movements to rehabilitation in virtual reality. Cortex. 2021 Nov;144:82-98. doi: 10.1016/j.cortex.2021.08.008. Epub 2021 Sep 24. PMID: 34662720.
16. Tieri G, Morone G, Paolucci S, Iosa M. Virtual reality in cognitive and motor rehabilitation: facts, fiction and fallacies. Expert Rev Med Devices. 2018 Feb;15(2):107-117. doi: 10.1080/17434440.2018.1425613. Epub 2018 Jan 10. PMID: 29313388.
17. Miterko LN, Baker KB, Beckinghausen J, Bradnam LV, Cheng MY, Cooperrider J, DeLong MR, Gornati SV, Hallett M, Heck DH, Hoebeek FE, Kouzani AZ, Kuo SH, Louis ED, Machado A, Manto M, McCambridge AB, Nitsche MA, Taib NOB, Popa T, Tanaka M, Timmann D, Steinberg GK, Wang EH, Wichmann T, Xie T, Sillitoe RV. Consensus Paper: Experimental Neurostimulation of the Cerebellum. Cerebellum. 2019 Dec;18(6):1064-1097. doi: 10.1007/s12311-019-01041-5. PMID: 31165428; PMCID: PMC6867990.
18. Cattaneo Z, Ferrari C, Ciricugno A, Heleven E, Schutter DJLG, Manto M, Van Overwalle F. New Horizons on Non-invasive Brain Stimulation of the Social and Affective Cerebellum. Cerebellum. 2022 Jun;21(3):482-496. doi: 10.1007/s12311-021-01300-4. Epub 2021 Jul 16. PMID: 34270081.
19. Oldrati V, Ferrari E, Butti N, Cattaneo Z, Borgatti R, Urgesi C, Finisguerra A. How social is the cerebellum? Exploring the effects of cerebellar transcranial direct current stimulation on the prediction of social and physical events. Brain Struct Funct. 2021 Apr;226(3):671-684. doi: 10.1007/s00429-020-02198-0. Epub 2021 Jan 11. PMID: 33426567.
20. Ferrari C, Ciricugno A, Urgesi C, Cattaneo Z. Cerebellar contribution to emotional body language perception: a TMS study. Soc Cogn Affect Neurosci. 2019 Oct 7;17(1):81–90. doi: 10.1093/scan/nsz074. Epub ahead of print. PMID: 31588511; PMCID: PMC8824541.
21. Ciricugno A, Ferrari C, Rusconi ML, Cattaneo Z. The left posterior cerebellum is involved in orienting attention along the mental number line: An online-TMS study. Neuropsychologia. 2020 Jun;143:107497. doi: 10.1016/j.neuropsychologia.2020.107497. Epub 2020 May 12. PMID: 32413432.
22. Ferrari C, Ciricugno A, Battelli L, Grossman ED, Cattaneo Z. Distinct Cerebellar regions for Body Motion Discrimination. Soc Cogn Affect Neurosci. 2019 Dec 10;17(1):72–80. doi: 10.1093/scan/nsz088. Epub ahead of print. PMID: 31820788; PMCID: PMC8824544.
23. Oldrati V, Schutter DJLG. Targeting the Human Cerebellum with Transcranial Direct Current Stimulation to Modulate Behavior: a Meta-Analysis. Cerebellum. 2018 Apr;17(2):228-236. doi: 10.1007/s12311-017-0877-2. PMID: 28786014; PMCID: PMC5849643.

24. Thut G, Bergmann TO, Fröhlich F, Soekadar SR, Brittain JS, Valero-Cabré A, Sack AT, Miniussi C, Antal A, Siebner HR, Ziemann U, Herrmann CS. Guiding transcranial brain stimulation by EEG/MEG to interact with ongoing brain activity and associated functions: A position paper. Clin Neurophysiol. 2017 May;128(5):843-857. doi: 10.1016/j.clinph.2017.01.003. Epub 2017 Jan 29. PMID: 28233641; PMCID: PMC5385293.
25. Miyaguchi S, Otsuru N, Kojima S, Yokota H, Saito K, Inukai Y, Onishi H. The effect of gamma tACS over the M1 region and cerebellar hemisphere does not depend on current intensity. J Clin Neurosci. 2019 Jul;65:54-58. doi: 10.1016/j.jocn.2019.03.045. Epub 2019 Apr 3. PMID: 30954355.
26. Naro A, Leo A, Russo M, Cannavò A, Milardi D, Bramanti P, Calabrò RS. Does Transcranial Alternating Current Stimulation Induce Cerebellum Plasticity? Feasibility, Safety and Efficacy of a Novel Electrophysiological Approach. Brain Stimul. 2016 May-Jun;9(3):388-395. doi: 10.1016/j.brs.2016.02.005. Epub 2016 Feb 12. PMID: 26946958.
27. Naro A, Bramanti A, Leo A, Manuli A, Sciarrone F, Russo M, Bramanti P, Calabrò RS. Effects of cerebellar transcranial alternating current stimulation on motor cortex excitability and motor function. Brain Struct Funct. 2017 Aug;222(6):2891-2906. doi: 10.1007/s00429-016-1355-1. Epub 2017 Jan 7. PMID: 28064346.
28. De Zeeuw CI, Hoebeek FE, Schonewille M. Causes and consequences of oscillations in the cerebellar cortex. Neuron. 2008 Jun 12;58(5):655-8. doi: 10.1016/j.neuron.2008.05.019. PMID: 18549777.
29. D'Angelo E, Nieus T, Maffei A, Armano S, Rossi P, Taglietti V, Fontana A, Naldi G. Theta-frequency bursting and resonance in cerebellar granule cells: experimental evidence and modeling of a slow k+-dependent mechanism. J Neurosci. 2001 Feb 1;21(3):759-70. doi: 10.1523/JNEUROSCI.21-03-00759.2001. PMID: 11157062; PMCID: PMC6762330.
30. Naro A, Milardi D, Cacciola A, Russo M, Sciarrone F, La Rosa G, Bramanti A, Bramanti P, Calabrò RS. What Do We Know About the Influence of the Cerebellum on Walking Ability? Promising Findings from Transcranial Alternating Current Stimulation. Cerebellum. 2017 Aug;16(4):859-867. doi: 10.1007/s12311-017-0859-4. PMID: 28456901.
31. Heise KF, Kortzorg N, Saturnino GB, Fujiyama H, Cuypers K, Thielscher A, Swinnen SP. Evaluation of a Modified High-Definition Electrode Montage for Transcranial Alternating Current Stimulation (tACS) of Pre-Central Areas. Brain Stimul. 2016 Sep-Oct;9(5):700-704. doi: 10.1016/j.brs.2016.04.009. Epub 2016 Apr 14. PMID: 27160465.
32. Di Russo F, M B, V B, Rl P, S P, F Q, D S. Normative event-related potentials from sensory and cognitive tasks reveal occipital and frontal activities prior and following visual events. Neuroimage. 2019 Aug 1;196:173-187. doi: 10.1016/j.neuroimage.2019.04.033. Epub 2019 Apr 11. PMID: 30981857.
